# Integrated Evaluation of *Urtica dioica* Extract Assessing Physiochemical Analysis with Antioxidant, Antiviral, and Immunomodulatory Effects Against SARS-CoV-2

**DOI:** 10.3390/ph19050693

**Published:** 2026-04-28

**Authors:** Gulsah Akbas, Seyma Aydinlik, Jenya Dursun, Frederick Lia, Mustafa Emrem, Banu Mansuroğlu, Yuksel Cetin

**Affiliations:** 1 Life Sciences Unit, Marmara Research Center, The Scientific and Technological Research Council of Turkey (TUBITAK), Gebze 41470, Turkey; gulsah.akbas34@gmail.com (G.A.); seyma.aydinlik@tubitak.gov.tr (S.A.); jenyadursun@gmail.com (J.D.); memrem29@gmail.com (M.E.); 2 Department of Molecular Biology and Genetics, Faculty of Arts and Sciences, Yildiz Technical University, Istanbul 34220, Turkey; banumansur@gmail.com; 3 Chemistry Group Laboratories, TUBITAK UME, Gebze 41470, Turkey; 4 Institute of Applied Sciences, Malta College of Arts, Science and Technology, Paola PLA9032, Malta; frederick.lia@mcast.edu.me

**Keywords:** *Urtica dioica* extract, SARS-CoV-2, antiviral activity, antioxidant activity, immunomodulatory effect, RBD-ACE2 binding inhibitor, Mpro inhibitor

## Abstract

**Background:** A major challenge in antiviral development is the identification of novel virus–host interactions while ensuring therapeutic efficacy and safety. These challenges have renewed interest in phytochemicals derived from medicinal plants as alternative antiviral agents. **Objectives:** In this study, we investigated the antioxidant, antiviral, and immunomodulatory properties of a Mediterranean *Urtica dioica* extract (UdE) against SARS-CoV-2 using chemical, biochemical, and *in vitro* approaches. **Methods:** The physicochemical properties of UdE were characterized using microtiter assays and HPLC analysis. Cytocompatibility was evaluated in HEK293T, Vero E6, Caco-2, and Calu-3 cell lines while antioxidant activity was assessed using both chemical and cell-based assays. Antiviral activity was evaluated by assessing inhibition of SARS-CoV-2 receptor binding domain (RBD)–ACE2 interaction using ELISA, inhibition of SARS-CoV-2 main protease (Mpro) activity via FRET assay and inhibition of viral entry using SARS-CoV-2 S1 pseudovirus neutralization assay. **Results:** UdE (100 µg/mL) inhibited RBD–ACE2 binding by 94% and suppressed Mpro activity by 74%, while reducing moderate but significant inhibition of pseudovirus entry (33.6%) at 300 µg/mL dose level in ACE2 expressing HEK293T cells. Immunomodulatory analysis revealed significant suppression of IL-1β and IL-6 production, accompanied by increased TNF-α and IL-8 levels. **Conclusions:** Collectively, these findings highlight that UdE exhibits multi-target *in vitro* antioxidant, antiviral, and immunomodulatory activity against SARS-CoV-2; therefore, UdE represents a promising bioactive extract for the management of SARS-CoV-2 infection.

## 1. Introduction

The pandemic highlighted limitations of single target antiviral approaches. Antiviral efficacy was often influenced by viral mutations, timing of administration, and host-related factors such as age, comorbidities, and immune status. This complexity increased interest in adjunct and alternative therapeutic strategies, including host-directed therapies, immunomodulators, antioxidants, and natural products, which could complement conventional antivirals by targeting inflammation, oxidative stress, and immune dysregulation rather than the virus alone. This conceptual framework has shifted antiviral research beyond purely virus directed inhibition toward host directed and multitarget strategies that aim to interfere with critical viral processes while preserving cellular homeostasis and limiting inflammation associated damage [1,2]. Another major advantage of host-directed inhibition over virus-directed inhibition is that the risk of mutations occurring and thus resistance developing during treatment is much lower. Within this context, phenolic-rich natural products have attracted renewed attention as sources of multi-functional bioactivity. Viral attachment and entry are initiated by the interaction between the receptor binding domain (RBD) of the spike glycoprotein and the host angiotensin-converting enzyme 2 (ACE2) receptor, followed by protease dependent activation and membrane fusion [3,4]. Disruption of the RBD–ACE2 interface therefore represents a validated antiviral intervention point, while downstream processing of viral polyproteins by the main protease (3CLpro/Mpro) remains essential for viral replication and maturation [5]. Consequently, strategies capable of simultaneously interfering with entry-associated mechanisms and replication-associated protease activity provide a rational framework for multi-target antiviral intervention.

Beyond direct viral targeting, increasing evidence indicates that host redox balance and inflammatory signaling play central roles in shaping infection outcomes. Excessive oxidative stress can amplify inflammatory cascades and impair effective antiviral im-mune responses, thereby contributing to tissue damage and disease severity [6]. Within this context, interventions capable of modulating oxidative and inflammatory pathways may indirectly influence viral pathogenesis. In particular, the nuclear factor erythroid 2–related factor 2 (*Nrf2*)–heme oxygenase-1 (*HO-1*) axis represents a key cytoprotective pathway that regulates cellular redox homeostasis and inflammatory responses. Activation of Nrf2 promotes the transcription of downstream antioxidant genes, including HO-1, thereby attenuating oxidative stress-induced damage and suppressing pro-inflammatory signaling cascades [7,8]. Moreover, recent studies suggest that pharmacological or natural activation of the *Nrf2/HO-1* pathway may confer antiviral benefits by limiting virus-induced oxidative injury and modulating host immune responses, highlighting its relevance as a supportive target in antiviral strategies [9]. Phenolic-rich natural products, characterized by their redox-active and immunomodulatory properties, have therefore attracted renewed attention as potential multi-functional agents in antiviral research. *Urtica dioica* L. (stinging nettle) is a medicinal plant with a long history of use and an expanding body of pharmacological evidence supporting its antioxidant, anti-inflammatory, and immunomodulatory properties [10]. Contemporary phytochemical analyses consistently describe UdE as rich in hydroxycinnamic acids and related phenolics, with chlorogenic acid frequently identified as a major constituent [11,12]. These compounds are known to modulate redox homeostasis and inflammatory signaling pathways, thereby contributing to the biological activity of phenolic-rich plant extracts [13,14]. In addition, chlorogenic acid, a phenolic compound found in UdE, has shown anti-SARS-CoV-2 activity *in vitro* in other plant sources exhibited inhibitory activity against SARS-CoV-2 spike–ACE2 interactions in binding assays, with variant-dependent potency [15,16]. Therefore, these findings suggest that the components of *Urtica dioica* may collectively contribute to antiviral effects through complementary mechanisms, highlighting the need for holistic experimental evaluation targeting different viral and host-related objectives.

While these reductionist approaches have provided valuable mechanistic insights into specific entry-related processes, they do not capture the complexity of the phytochemical matrix present in the native plant. Therefore, the present study aims to systematically evaluate the antioxidant, antiviral, and immunomodulatory effects of UdE using an integrated framework combining physicochemical analysis, biochemical assays, and *in vitro* cell-based models targeting of SARS-CoV-2 entry, Mpro activity and host inflammatory responses.

## 2. Results

### 2.1. Physicochemical Analysis of UdE

#### 2.1.1. Microtiter Quantification

Microtiter based quantification of UdE was performed to determine its total phenolic content (TPC), total flavonoid content (TFC), flavanol content, total ortho-diphenolic content (TdOPC), and aucubin content. The results are expressed as gallic acid equivalents (GAE), quercetin equivalents (QE), pyrocatechol equivalents (PE), and aucubin equivalents (AE), respectively, and reported as mg/g of extract. The external standard calibration curves of the quantified TPC, TFC, and TdOPC are presented in the Supplementary Materials (Figures S1–S3). UdE exhibited a TPC of 11.37 ± 2.18 mg GAE/g, a TFC of 195.07 ± 28.55 mg QE/g, a flavanol content of 15.53 ± 0.36 mg QE/g, and a TdOPC of 113.74 ± 8.63 mg PE/g. In addition, the aucubin content was determined to be 1.58 ± 0.10 mg AE/g. Collectively, these findings demonstrate that UdE possesses a phytochemical profile enriched in polyphenolic and flavonoid compounds, which may contribute to its observed biological activities.

#### 2.1.2. High Performance Liquid Chromatography (HPLC)

**Figure 1:**
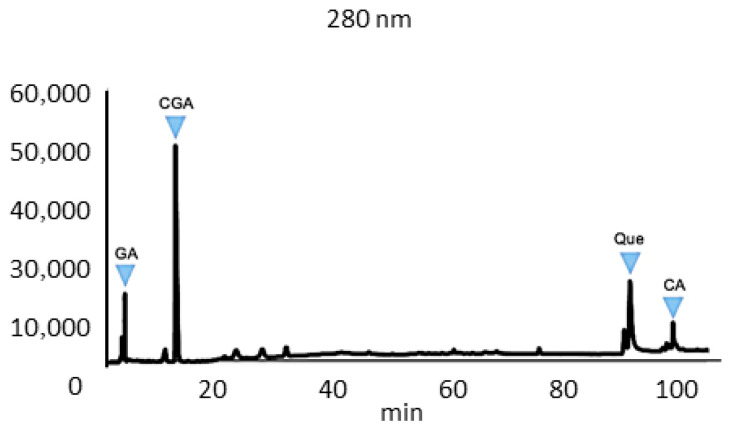
HPLC chromatographic profiles of UdE at 280 nm. Peaks corresponding to key phenolic constituents are annotated: gallic acid (GA; tR = 3 min; LOD = 0.266 µg/mL; LOQ = 0.80 µg/mL), chlorogenic acid (CGA; tR = 12 min; LOD = 0.505 µg/mL; LOQ = 1.53 µg/mL), quercetin (Que; tR = 97 min; LOD = 0.586 µg/mL; LOQ = 1.77 µg/mL) and caffeic acid (CA; tR = 88 min; LOD = 0.600 µg/mL; LOQ = 1.82 µg/mL). Retention times (tR) are reported in minutes; LOD and LOQ values are reported as calculated for the method.

Quantitative profiling of phenolic and flavonoid constituents in UdE was performed using a rigorously validated HPLC (Figure 1). The calibration curves were constructed for fifteen analytical reference standards—gallic acid, chlorogenic acid, caffeic acid, vanillic acid, p-coumaric acid, vanillin, ferulic acid, 2-hydroxycinnamic acid, rosmarinic acid, quercetin, apigenin, kaempferol, hesperetin, 5,7-dihydroxyflavone, and rutin—each exhibiting excellent linearity within the optimized concentration range, as indicated by correlation coefficients (R^2^) exceeding 0.998 (Supplementary Materials, Table S1, Figure S4a,b). The HPLC chromatogram of the standards are given in the Supplementary Materials (Figure S5).

The method validation encompassed assessment of precision, linearity, and analytical sensitivity. The limits of detection (LOD) and quantification (LOQ) were derived demonstrating the method’s high sensitivity and reproducibility. These validation metrics confirm the robustness and analytical reliability of the developed HPLC procedure for the comprehensive characterization of UdE-derived polyphenols. HPLC analysis revealed a distinct phenolic profile for the UdE, characterized by the predominance of hydroxycinnamic and hydroxybenzoic acid derivatives. Among the identified compounds, chlorogenic acid emerged as the most abundant phenolic constituent (45.9 ± 6.1 mg/g), followed by gallic acid (9.05 ± 1.5 mg/g), quercetin (6.6 ± 1.0 µg/g), caffeic acid (4.65 ± 0.8 mg/g), vanillic acid (2.5 ± 0.3 mg/g) and rosmarinic acid (1.2 ± 0.1 mg/g) in dry weight. UdE included trace amount of ferulic acid (0.4 mg/g), p-coumaric Acid (0.3 mg/g), 2-hydroxycinnamic acid (0.2 mg/g), hesperetin (0.2 mg/g) and rutin (0.1 mg/g). However, apigenin, kaempferol, and 5,7-dihydroxyflavone were not detected within the analytical range. The phytochemical profile of UdE consisted predominantly of phenolic acids, including chlorogenic, gallic, caffeic, vanillic, and rosmarinic acids, with flavonoids such as quercetin, hesperetin, and rutin present at lower concentrations. These findings indicate that the extract is enriched in non-flavonoid phenolics, with flavonoids representing a smaller fraction of the identified compounds.

### 2.2. UdE Effects on Cell Viability, Morphology, and Apoptotic Features

**Figure 2:**
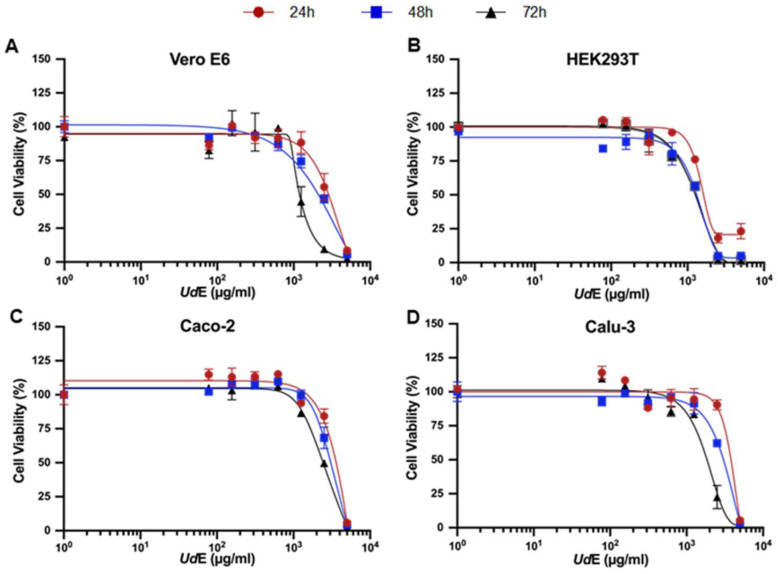
The dose–response relationship of UdE with (**A**) Vero E6, (**B**) HEK293T, (**C**) Caco-2, (**D**) Calu-3 cell lines upon 24, 48 and 72 h exposure determined by MTT cell viability assay. Data are presented as mean ± SD of 3 independent biological replicates.

Cell Viability: The dose- and time-dependent effects of UdE on the viability of Vero E6, HEK293T, Calu-3, and Caco-2 cell monolayers following 24, 48, and 72 h of exposure were evaluated using the MTT assay (Figure 2). As shown in Figure 2, UdE treatment resulted in a progressive reduction in cell viability in a dose- and time-dependent manner, and the corresponding TD_30_ and TD_50_ values were calculated using GraphPad Prism 8.2 software (Table 1). Across all cell lines, UdE induced a concentration-dependent decrease in cell viability that became more pronounced with prolonged exposure, particularly at 48 and 72 h compared with 24 h. Among the tested cell lines, HEK293T cells exhibited the highest sensitivity to UdE exposure, followed in descending order by Vero E6, Caco-2, and Calu-3 cells. Based on these cytotoxicity profiles, UdE concentrations below the TD_30_ values were selected for subsequent experiments, including morphological assessment, antioxidant activity analysis, and SARS-CoV-2 S1 pseudovirus neutralization assay.

**Figure 3:**
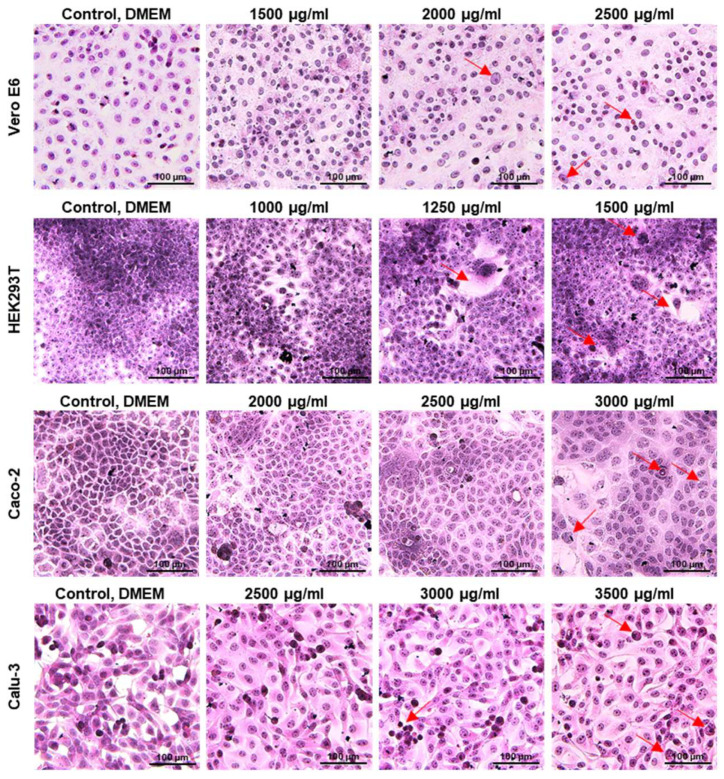
The effect of UdE on cell morphology after 24 h of application on Vero E6, HEK293T, Caco-2 and Calu-3 cell lines was evaluated by hematoxylin–eosin staining. Cytoplasmic vacuolization, cell swelling, loss of cell–cell adhesion, and nuclear deformation are shown with red arrows. Photos for each cell line and treatment are presented within the 3 independent biological replicates with 40× magnification, scale bar 100 µm.

Cell Morphology: Comparative analysis of hematoxylin and eosin (H&E) stained cell monolayers following 24 h of UdE exposure revealed distinct, cell line specific morphological responses (Figure 3). Among the tested cell lines, HEK293T cells exhibited clear dose-dependent morphological changes following extract exposure. Increasing concentrations were associated with progressive disruption of cell integrity, including cytoplasmic vacuolization and swelling, reduced cell adhesion, and marked nuclear deformation, indicative of heightened cellular stress. At higher doses, these alterations became more prominent, Comparatively, Vero E6 cells displayed intermediate sensitivity, characterized by moderate loss of cell–cell contacts and increased intercellular spaces with increasing UdE doses, while largely preserving overall cellular architecture at lower concentrations. Calu-3 and Caco-2 cells showed minimal morphological changes under the same treatment conditions, maintaining a more intact monolayer and cellular morphology across the tested concentration range. Overall, these observations are consistent with the cytotoxicity profiles obtained from the MTT assay.

**Figure 4:**
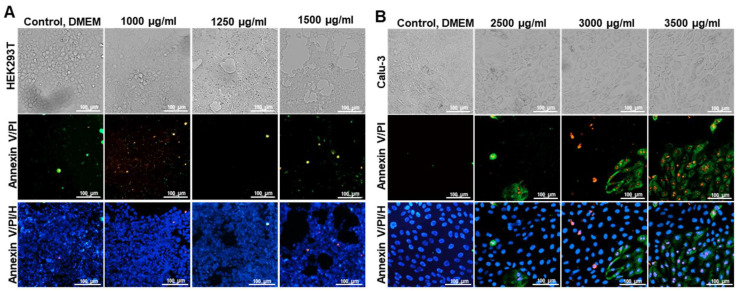
The apoptotic effects of UdE on upon application on HEK293T (**A**) and Calu-3 (**B**) for 24 h was evaluated using Annexin V (green), PI (red) and Hoechst (blue) staining. Images were taken at 40× magnification, scale bar 100 µm.

Apoptotic Features: Apoptotic effects of UdE on Calu-3 and HEK293T cells was examined by Annexin V, PI and Hoechts staining and the images were taken with a fluorescence microscope. Annexin V-FITC, propidium iodide (PI), and Hoechst staining of the cells showed that apoptotic and necrotic changes in the cell lines were observed in a dose-dependent manner characterized by red and green staining in the images (Figure 4). Upon exposure to increasing concentrations of UdE, HEK293T cells displayed early apoptotic characteristics, including mild cell shrinkage, loss of adherence, and a gradual increase in apoptotic signal intensity. In contrast, Calu-3 cells exhibited a more pronounced and concentration-dependent apoptotic response. Even at intermediate UdE doses, marked morphological alterations were observed, including membrane blebbing, cell rounding, and disruption of the epithelial monolayer. The apparent discrepancy between the apoptosis data and cell viability assays in Calu-3 cells can be attributed to differences in assay sensitivity and biological endpoints. While apoptosis assays detect early cellular stress responses, MTT-based viability measurements primarily reflect later-stage metabolic impairment. Therefore, the presence of apoptotic features without a pronounced decrease in viability suggests that UdE induces early, sublethal cellular stress in Calu-3 cells rather than immediate cytotoxicity. In untreated control groups, both cell lines exhibited preserved epithelial morphology, intact cell–cell contacts, and homogeneous nuclear staining, indicative of healthy and viable monolayers.

### 2.3. Chemical and Cellular Based Antioxidant Activity of UdE

**Figure 5:**
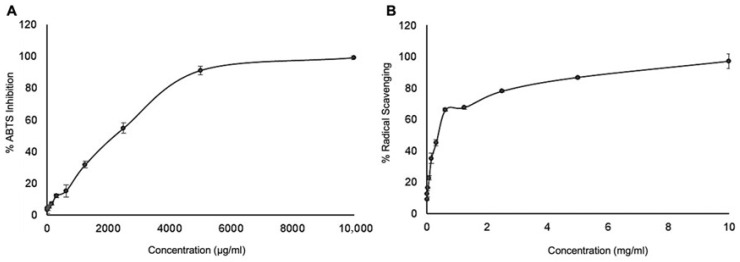
The percentage of radical-scavenging efficiency of UdE, determined through (**A**) ABTS and (**B**) DPPH assays.

To provide a comprehensive evaluation of UdE, electron transfer based ferric reducing antioxidant power (FRAP) and cupric reducing antioxidant power (CUPRAC) assays assessed, while hydrogen peroxide scavenging activity (H_2_O_2_) and 2,2-Diphenyl-1-picrylhydrazyl (DPPH) evaluated, hydrogen atom transfer based mediated radical scavenging; 2,2′-azino-bis(3-ethylbenzothiazoline-6-sulfonic acid) (ABTS) reflected a mixed electron and hydrogen transfer mechanism assays were performed. The percentage of radical-scavenging efficiency of UdE, determined through ABTS and DPPH assays were found as 1375.71 ± 33.66 µg/mL and 0.35 ± 0.04 mg/mL, respectively (Figure 5A,B). Both assays revealed a concentration dependent increase in scavenging activity, with the extract exhibiting notably higher ABTS% inhibition compared to DPPH. The antioxidant activities of the extracts were quantitatively assessed using FRAP, CUPRAC, and H_2_O_2_ scavenging assays to evaluate their total reducing and radical-neutralizing capacities. Among the tested samples, UdE exhibited the highest FRAP (824.20 ± 107.13 mg AAE/g) followed by notable CUPRAC (309.72 ± 193.17 mg GAE/g), and H_2_O_2_ scavenging (7.41 ± 1.24 mg AAE/g) activities. These findings indicated an electron-donating potential and effective ROS suppression of UdE, reflecting the high phenolic density and synergistic antioxidant interactions within the extract.

**Figure 6:**
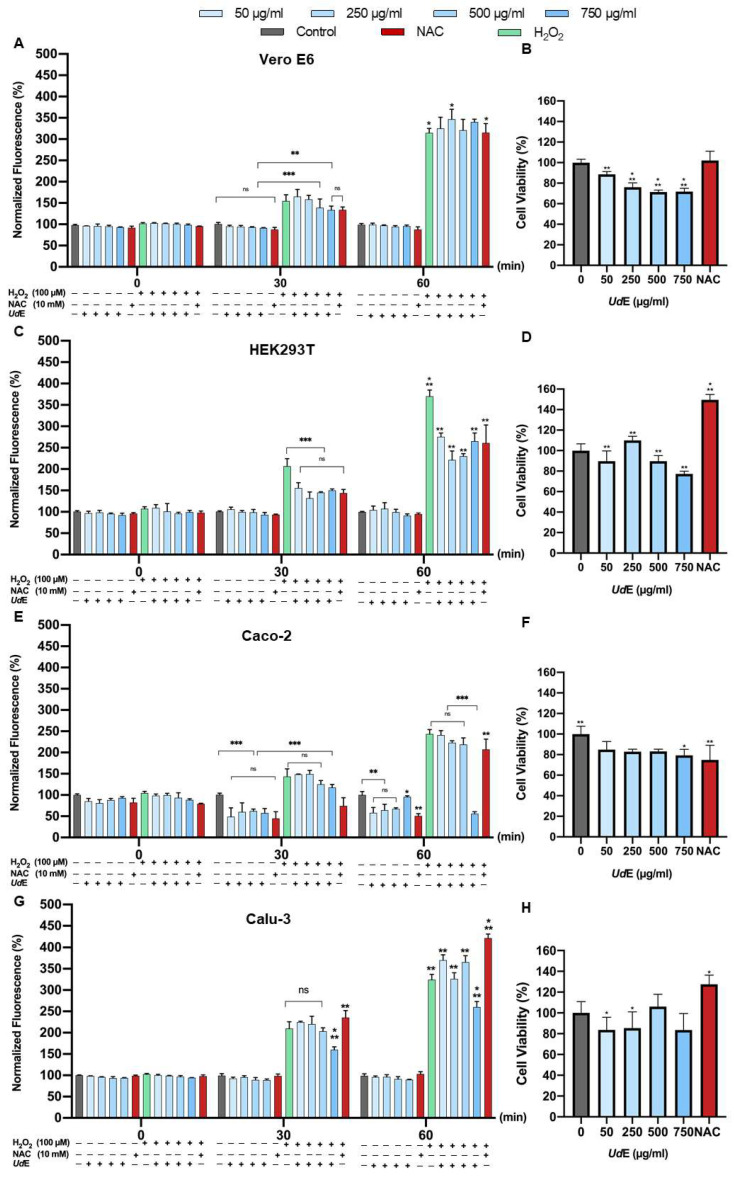
The cellular based antioxidant activity of UdE was evaluated in (**A**) Vero E6, (**C**) HEK293T, (**E**) Caco-2 and (**G**) Calu-3, cell lines at concentrations ranged from 50 to 750 µg/mL using DCFDA assay. In parallel, (**B**,**D**,**F**,**H**) cell viability of the tested cells was evaluated at the same conditions by MTT assay. Data are presented as mean ± SD of 3 independent biological replicates. Statistical significance was determined using two-way ANOVA followed by Tukey’s multiple comparisons test (* *p* < 0.05, ** *p* < 0.01, *** *p* < 0.001, ns = not significant).

The antioxidant activity of UdE was evaluated by performing cellular based DCFDA assay using Vero E6, HEK293T, Caco-2, and Calu-3 cell lines at concentrations ranged from 50 to 750 µg/mL (Figure 6). In parallel, cell viability of the examined cell lines was assessed under the same conditions and all tested concentrations maintained ≥75% viability relative to untreated controls indicating that the observed effects were not associated with significant cytotoxicity. Under H_2_O_2_-induced oxidative stress conditions, UdE treatment resulted in a significant reduction in intracellular ROS levels in a concentration-dependent manner. UdE at 750 µg/mL significantly reduced oxidative stress in HEK293T, Caco-2 and Calu-3 cell lines compared to the H_2_O_2_-treated control (*p* < 0.01). UdE at 750 µg/mL significantly reduced ROS levels in Caco-2 and Calu-3 cell lines more than NAC-treated cells particularly hydrogen peroxide treated conditions (*p* < 0.01) whereas it was as effective as NAC in HEK293T cells. The extract exhibited a more pronounced radical scavenging effect in Caco-2 and Calu-3 cells more than NAC which is a well-known antioxidant used as a positive control, in the presence of H_2_O_2_ although the magnitude of reduction was effective only in Calu-3 cells during the very early time points of the experiment. In comparison, HEK293T, Caco-2, and Calu-3 cell monolayers exhibited higher levels of antioxidative response compared with Vero E6 cells under H_2_O_2_-induced conditions.

**Figure 7:**
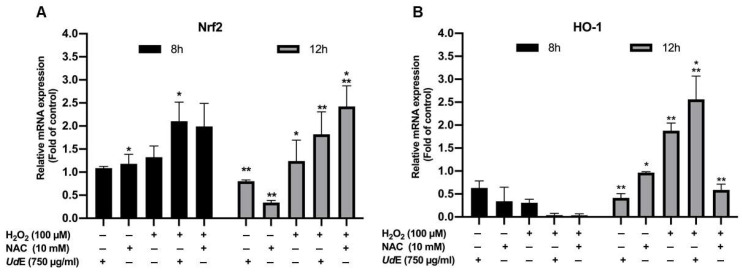
HEK293T cells were treated with UdE (750 µg/mL) under H_2_O_2_-induced oxidative stress, and (**A**) *Nrf2* and (**B**) *HO-1* mRNA levels were quantified by RT-qPCR. Expression levels were normalized to *GAPDH* and calculated using the 2^−ΔΔCt^ method. Data are presented as mean ± SEM from three independent biological replicates. Statistical significance was determined using two-way ANOVA followed by Tukey’s multiple comparisons test (* *p* < 0.05, ** *p* < 0.01).

To investigate the molecular basis of the antioxidant activity of UdE, the expression levels of the nuclear factor erythroid 2-related factor 2 (*Nrf2*) and its downstream target heme oxygenase-1 (*HO-1*) were evaluated in HEK293T cells using RT-qPCR (Figure 7). UdE treatment resulted in a significant upregulation of *Nrf2* expression under H_2_O_2_-induced oxidative stress conditions. This increase was observed at both time points, with a more pronounced effect at the later time point (*p* < 0.01), indicating a time-dependent activation of *Nrf2* signaling. Similarly, *HO-1* expression was significantly elevated following UdE treatment, consistent with the activation of downstream antioxidant defense mechanisms (*p* < 0.01). Notably, the magnitude of *HO-1* induction was greater than that of *Nrf2*, suggesting effective transcriptional activation of *Nrf2* target genes. In comparison to the oxidative stress control, UdE treatment restored or enhanced the expression levels of both *Nrf2* and *HO-1*, demonstrating its capacity to modulate key antioxidant pathways at the transcriptional level.

### 2.4. Chemical and Cellular Based Antiviral Activity of UdE

#### 2.4.1. RBD-ACE2 Binding and Mpro Activity Inhibiton Potential of UdE

**Figure 8:**
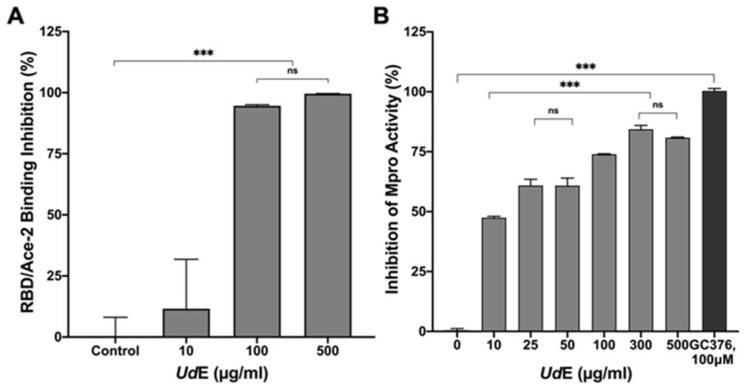
Dual-platform assessment of UdE–mediated inhibition potential against SARS-CoV-2. (**A**) RBD-ACE2 binding inhibitory potential of UdE in a concentration-dependent reduction (**B**) Inhibition effect on UdE on Mpro (Negative control: Mpro; positive control: GC367). Data are presented as means ± SD, (*n* = 3) Statistical significance was determined using one-way ANOVA followed by Tukey’s multiple comparisons test (*** *p* < 0.001, ns = not significant).

RBD-ACE2 binding inhibition of UdE: Antiviral potential of UdE against SARS-CoV-2 was investigated at the RBD–ACE2 binding interface which represent critical nodes in viral attachment and entry to host cell. RBD-ACE2 binding inhibitory screening revealed that UdE exhibited a dose-dependent inhibitory capacity with 25.90%, 94.28% and 99.57% observed at concentrations of 10 µg/mL, 100 µg/mL and 500 µg/mL, respectively. The obtained 50% of inhibitory concentration (EC_50_) of UdE was calculated as 50.14 ± 11.91 µg/mL (Figure 8A). To clarify the ELISA assay limitations for polyphenol-rich extract that can interfere with protein–protein interaction assays through nonspecific binding, redox activity, or aggregation effects, the RBD–ACE2 ELISA assay provides a preliminary indication of interaction interference and should not be interpreted as definitive evidence of direct antiviral activity. SARS-CoV-2 Mpro inhibition of UdE: The inhibitory potential of UdE on SARS-CoV-2 Mpro activity was examined to determine whether UdE can impede viral replication following vital entry into host cells. Accordingly, the inhibitory effect of UdE against Mpro activity at concentrations of 10, 25, 50, 100, 300, and 500 µg/mL was determined 47.49%, 60.89%, 60.83%, 73.94%, 84.34% and 80.79%, respectively as compared with positive control GC365 (100 µM), which exhibited 100% Mpro inhibition (Figure 8B). The EC_50_ value of UdE was calculated as as 12.88 ± 1.06 µg/mL with GraphPad Prism version 8.2. These findings demonstrate that UdE directly interferes with Mpro activity, supporting its potential role in inhibiting viral replication at the post-entry stage.

#### 2.4.2. SARS-CoV-2 S1 Pseudovirus Neutralization Potential of UdE

**Figure 9:**
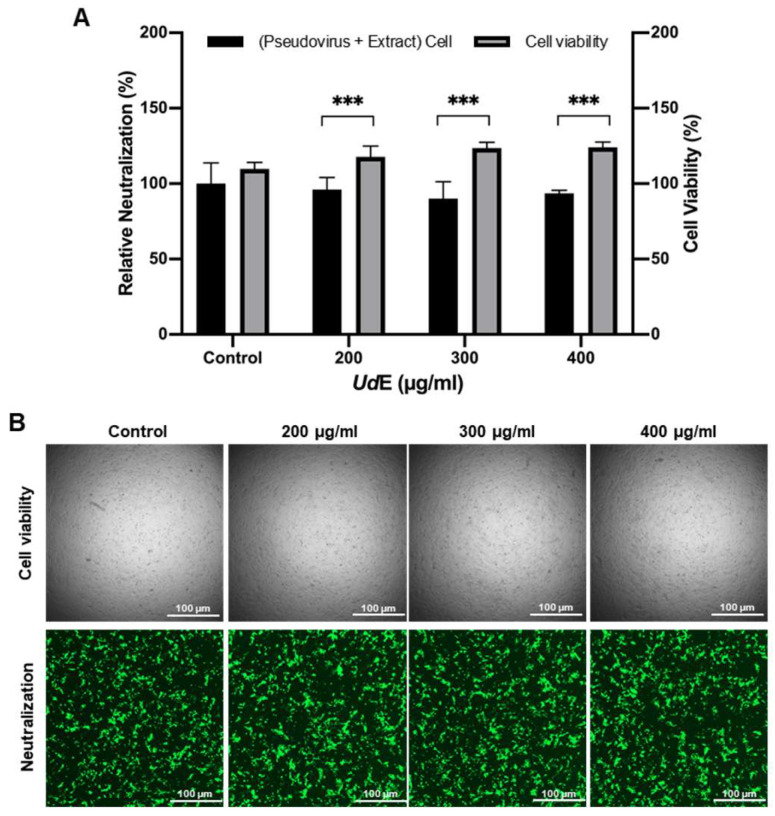
Dose-dependent inhibition of SARS-CoV-2 S1 pseudovirus entry by UdE in ACE2-transfected HEK293T cells. (**A**) Relative fluorescence-based pseudovirus neutralization following treatment for 72 h with increasing concentrations of UdE and cell viability (**B**) Bright-field and fluorescence images of HEK293T cells under the same experimental conditions. Data are presented as means ± SD, (*n* = 3). Differences between groups were evaluated by one-way analysis of variance (ANOVA) followed by Tukey’s multiple comparisons test, *** *p* < 0.001. Images were taken with 4x magnification, scale bar 1000 µm.

The potential of SARS-CoV-2 S1 pseudovirus protein neutralizing activity of the UdE was investigated using ACE2 transfected HEK293T cells with increased ACE2 expression. The neutralization potential of UdE at concentrations of 200, 300, and 400 µg/mL resulted in reductions of viral signal by 25.65%, 33.60%, and 27.91%, respectively. When viral readouts were normalized to the cell viability determined by the CellTiter-Glo assay, UdE treatment resulted in a cell-normalized reduction in viral signal of 19.86%, 24.78%, and 20.50% at concentrations of 200, 300, and 400, respectively (Figure 9). Although the reduction of S1 pseudovirus entry to the host cell via UdE did not increase monotonically with dose, such non-linear fluctuations are commonly observed in cell-based assays and likely reflect biological variability and differences in host-cell metabolic responses rather than a loss of antiviral efficacy (*p* < 0.001).

### 2.5. Immunomodulatory Effects of UdE

**Figure 10:**
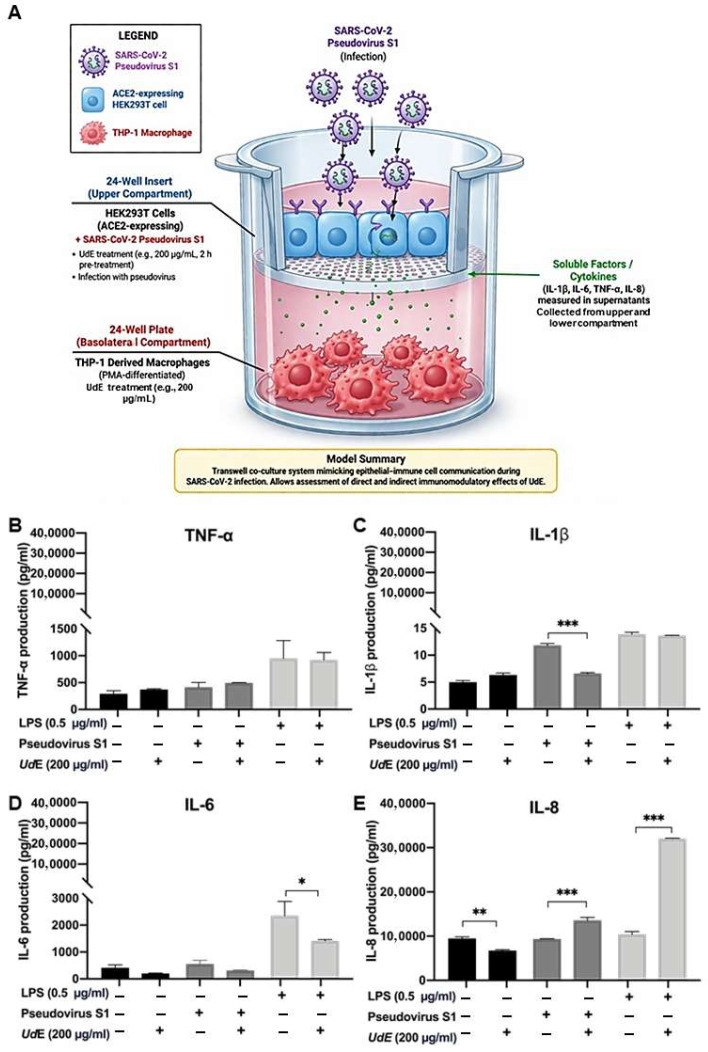
(**A**) Transwell co-culture system mimicking epithelial–immune cell communication during SARS-CoV-2 S1 pseudovirus infection (drawn via ChatGPT (OpenAl) based on GPT-5.3 model). Immunomodulatory effect of UdE (200 µg/mL) on (**B**) TNF-α, (**C**) IL-1β, (**D**) IL-6, and (**E**) IL-8 cytokine levels was evaluated using co-culture model consisting of HEK293T cells with SARS-CoV-2 S1 pseudovirus transduced and THP-1 monocyte-derived macrophages by ELISA. Data represent means ± SD, (*n* = 3), * *p* < 0.05, ** *p* < 0.01, *** *p* < 0.001 compared to untreated control (one-way ANOVA with Tukey’s post hoc test).

One of the major health challenges during the COVID-19 pandemic was the development of a cytokine storm; therefore, immunosuppressive bioactive extracts gained considerable prominence. To evaluate immunomodulatory effects of UdE, a co-culture model consisting of HEK293T cells transduced with SARS-CoV-2 S1 pseudovirus and THP-1 monocyte-derived macrophages was used to examine TNF-α, IL-1β, IL-6, and IL-8 levels by ELISA (Figure 10). Co-culture of THP-1 and HEK293T cells infected with SARS-CoV-2 S1 pseudovirus upon treatment with UdE (200 µg/mL) responded as reduction in IL-1β and IL-6 may indicate suppression of excessive inflammatory signaling, the concomitant increase in TNF-α and IL-8 suggests activation of innate immune pathways, including immune cell recruitment and early antiviral responses. Cytokine analysis revealed a stimulus-dependent immunomodulatory profile of UdE. In an LPS-induced inflammatory environment, UdE treatment resulted in a reduction of IL-6 levels, whereas no statistically significant suppression of IL-6 was observed upon pseudovirus challenge. Although LPS is a well-established inducer of IL-8 via TLR4 signaling, the absence of a robust IL-8 response in this model may be attributed to the differentiation state of THP-1-derived macrophages, the influence of epithelial–immune cell interactions in the transwell co-culture system, and the temporal dynamics of cytokine secretion. These factors may shift the cytokine response toward alternative inflammatory mediators such as IL-1β and IL-6. Notably, IL-1β levels were selectively reduced under pseudovirus-treated conditions, while no marked suppression was detected in the LPS-only setting. This differential cytokine response indicates that UdE exerts context-dependent modulation of inflammatory signaling, characterized by preservation of chemokine-associated responses alongside selective attenuation of specific pro-inflammatory cytokines depending on the upstream stimulus.

## 3. Discussion

UdE has been widely described in the literature as a regulatory antioxidant and anti-inflammatory medicinal plant, with its biological activity largely attributed to phenolic constituents such as chlorogenic acid and quercetin. For these compounds, antiviral relevance has predominantly been inferred from *in silico* studies targeting SARS-CoV-2 proteins, while experimental validation at the cellular level has remained limited. The present study addresses this gap by providing direct *in vitro* evidence that UdEs exert integrated antioxidant, immunomodulatory, and antiviral effects. This study demonstrated that the antiviral potential of UdE from the Mediterranean region is primarily attributable to its phytochemical profile, which was consisted predominantly of phenolic acids, including chlorogenic, gallic, caffeic, vanillic, and rosmarinic acids, while flavonoids such as quercetin, hesperetin, and rutin are present at lower concentrations, together with additive and synergistic interactions among the identified bioactive compounds.

In addition to cytotoxicity assessment, histological and fluorescence-based analyses revealed preserved cellular morphology at non-toxic doses (<TD_30_). UdE did not produce significant apoptotic features in HEK293T cells, whereas Calu-3 cells exhibited apoptotic features at the TD_30_ dose, as shown in Figure 4. Under these conditions, UdE effectively reduced intracellular ROS levels following oxidative challenge, consistent with its phenolic rich composition highlighting the contribution of the phytochemical matrix rather than isolated constituents. This temporal profile is in close agreement with previous *in vitro* studies reporting antioxidant and cytoprotective effects of UdE under oxidative stress conditions [17,18]. The consistency of these observations across distinct cell types supports the notion that UdE exerts dose and cell type independent redox-modulating activity. The RT-qPCR findings provide important mechanistic insight into the antioxidant effects of UdE observed in the cellular ROS assays. The significant upregulation of *Nrf2* and *HO-1* indicates that UdE not only acts as a direct radical scavenger but also activates endogenous antioxidant defense systems. Previous study reported that plant-derived polyphenolic compounds, such as those present in UdE, are known to exert dual functions, including both direct antioxidant activity and modulation of redox-sensitive signaling pathways such as the Nrf2/ARE axis [19]. Consistently, polyphenol-rich extracts of UdE have been shown to reduce intracellular ROS levels while enhancing antioxidant enzyme activities and modulating inflammatory responses [12]. Nrf2 is a key regulator of cellular redox homeostasis, and its activation induces cytoprotective genes such as *HO-1*, which plays a critical role in mitigating oxidative stress. The time-dependent increase in *Nrf2* and *HO-1* expression correlates with the delayed but enhanced reduction in ROS levels, suggesting that the antioxidant activity of UdE is largely mediated through transcription-dependent cellular mechanisms rather than immediate chemical scavenging alone. The more pronounced induction of *HO-1* further supports efficient downstream signaling and amplification of the antioxidant response, consistent with the activity of polyphenolic compounds. Overall, these findings indicate that UdE functions as a modulator of redox homeostasis via activation of the Nrf2/HO-1 pathway, which may also contribute to its potential antiviral effects. However, further studies are required to confirm pathway-specific involvement and the contribution of individual phytochemicals. In the context of viral infection, maintenance of redox balance may represent an important supportive mechanism by which bioactive extract contributes to host resilience. ROS play a dual role during viral infections, contributing to antiviral signaling at physiological levels while promoting tissue damage, inflammation, and viral pathogenesis when excessively elevated [20]. Excessive oxidative stress has been associated with increased viral permissiveness and dysregulated immune responses, whereas controlled redox balance may preserve antiviral defense while limiting collateral damage [21,22]. Mechanistically, the controlled antioxidant profile observed here is consistent with the polyphenol-rich composition of UdE, as phenolic acids and flavonoids such as chlorogenic acid and quercetin are known to modulate intracellular ROS through radical scavenging, metal chelation, and regulation of mitochondrial redox homeostasis [23]. Recent *in vitro* studies have demonstrated that polyphenol-rich fractions of *Urtica dioica* significantly reduce the production of pro-inflammatory cytokines, including IL-1β and IL-6, particularly under inflammatory or oxidative stress conditions such as LPS or H_2_O_2_ stimulation. This anti-inflammatory effect is accompanied by a reduction in intracellular ROS levels and enhancement of antioxidant enzyme activities (e.g., SOD, CAT), indicating a tight coupling between redox regulation and cytokine modulation [23].Collectively, these findings suggest that UdE does not simply suppress ROS indiscriminately, but rather promotes a balanced redox state that may be advantageous in limiting oxidative damage while preserving redox-dependent host defense mechanisms relevant to viral susceptibility.

Mpro is an indispensable viral enzyme responsible for cleavage of viral polyproteins at no fewer than 11 conserved sites, and its inhibition has been repeatedly shown to abrogate viral replication [24]. In the present study, UdE demonstrated, concentration-dependent inhibition potential of Mpro, yielding an EC_50_ value of 12.88 ± 1.06 µg/mL across the tested range (10–500 µg/mL) whereas Narayan et al. reported EC_50_ of 12.88 µg/mL for UdE [25]. For a crude plant extract, this level of potency is notable and compares favorably with previously reported plant-derived Mpro inhibitors evaluated in biochemical assays. Mechanistic interpretation of this inhibition is supported by quantitative data from studies on individual phenolic constituents abundant in UdE. *In silico* molecular docking analyses supported the involvement of bioactive compounds in SARS-CoV-2 Mpro inhibition. Docking simulations performed against Mpro crystal structures 6LU7 and 2GBT predicted stable binding of chlorogenic acid within the catalytic pocket, with calculated binding energies of approximately −6.0 kcal/mol (6LU7) and −7.4 kcal/mol (2GBT) [26]. In both models, chlorogenic acid was positioned in proximity to the catalytic dyad region, forming hydrogen-bond interactions consistent with interference at the active site. Consistently, Sabet et al. reported a docking score of −7.57 kcal/mol for chlorogenic acid, with detailed interaction mapping revealing hydrogen-bond formation with key active-site residues His163, Ser144, Glu166, and Arg188, which are critically involved in substrate recognition and stabilization within the Mpro catalytic cleft [27]. This study supported the insilico findings through ELISA-based screening, demonstrating the high inhibitory potential of UdE against SARS-CoV-2 Mpro activity, which may be attributed in part to its abundant chlorogenic acid content (45.9 ± 6.1 µg/g). In this study, UdE also contained quercetin (6.6 ± 1.0 µg/g) which has been demonstrated that quercetin inhibits SARS-CoV-2 Mpro using a FRET-based enzymatic assay, with kinetic analysis revealing an intrinsic K_i_ of approximately 7 µM. Consistently, molecular docking and molecular dynamics simulations predicted favorable binding of quercetin within the Mpro active site, with binding free energies ranging from −7.5 to −7.2 kcal/mol [28]. In parallel, Bahun et al. reported that quercetin exhibited inhibitory activity against SARS-CoV-2 Mpro in enzymatic assays, with an TD_50_ of approximately 23 µM. Direct binding of these compounds to Mpro was further confirmed by surface plasmon resonance, supported by complementary molecular docking and molecular dynamics simulations with docking energies reaching −8.05 kcal/mol [29]. In this study, ELISA-based screening demonstrated the high inhibitory potential of UdE against SARS-CoV-2 Mpro activity, while complementary *in silico* docking analyses provided a coherent structure-based rationale for the observed potent Mpro inhibition.

Viral entry mediated by the interaction between the SARS-CoV-2 RBD and the host ACE2 receptor represents a critical and druggable step in infection. In our study, two complementary *in vitro* approaches were employed to evaluate the inhibitory effect of UdE on viral entry: (i) a competitive RBD–ACE2 ELISA assay and (ii) a spike S1–pseudotyped virus neutralization assay. UdE exhibited near-complete inhibition of RBD–ACE2 binding, reaching approximately 99% inhibition at 500 µg/mL, as determined using a competitive RBD–ACE2 ELISA-based binding assay. While this level of inhibition indicates a strong interaction-disrupting effect within the assay system, it is important to note that polyphenol-rich extracts are known to potentially interfere with ELISA-based protein–protein interaction assays through nonspecific binding, redox activity, or aggregation effects. Therefore, the RBD–ACE2 ELISA results should be interpreted as indicative of interaction interference rather than definitive evidence of direct antiviral activity. Nevertheless, the magnitude of inhibition was consistent with previous reports employing surrogate live-virus plaque reduction neutralization tests (PRNT) and competitive ELISA formats for measuring serum neutralizing antibodies over post vaccination, in which 80–95% inhibition of RBD–ACE2 interaction [30]. Despite the pronounced blockade of RBD–ACE2 binding, UdE produced a moderate but reproducible reduction of viral entry in the present spike S1–pseudotyped virus assay, achieving 27.91% inhibition at 400 µg/mL under *in vitro* conditions using a GFP reporter system. Importantly, such partial inhibition is not unexpected and has been widely reported in pseudovirus-based entry assays, which integrate multiple steps beyond receptor binding, including spike conformational rearrangement, membrane fusion, and post-binding entry processes. In the current experimental design, the pseudovirus was pre-incubated with UdE prior to exposure to cells. Therefore, the observed inhibitory effect may result from multiple, non-mutually exclusive mechanisms, including (i) direct interaction with viral particles (potential virucidal or structural interference effects) and/or (ii) modulation of spike–ACE2 binding and entry processes. As a result, the present data do not allow definitive discrimination between virucidal activity and host cell entry inhibition. In another study, Xiong et al. demonstrated that compounds producing high levels of inhibition in biochemical RBD–ACE2 interaction assays typically resulted in only moderate suppression of spike-pseudotyped viral entry, with reported inhibition levels in the range of approximately 30–50% [31]. Thus, the observed disparity between near-complete ELISA inhibition and moderate pseudovirus neutralization reflects intrinsic differences between assay formats rather than a lack of biological activity. Mechanistically, the entry-inhibitory activity observed for UdE is consistent with its polyphenol-rich composition, particularly the presence of chlorogenic acid, quercetin, and the other bioactive compounds. In a comprehensive experimental study, Hsieh et al. evaluated chlorogenic acid using both competitive trimeric spike–ACE2 ELISA and spike-pseudotyped viral entry systems, demonstrating interference potential against spike–ACE2 binding that translated into partial but significant suppression of pseudovirus entry in ACE2-expressing cells [16,32]. Notably, pseudovirus inhibition consistently remained below complete neutralization despite robust biochemical binding interference, indicating that chlorogenic acid primarily attenuates viral entry efficiency rather than fully blocking spike-mediated membrane fusion [33]. *In silico* studies further supported these observations, as molecular docking analyses have consistently predicted favorable binding of chlorogenic acid to ACE2 and spike-associated interfaces, with reported binding energies typically ranging between −7 and −8 kcal/mol [16]. Several studies have attributed inhibition of spike-mediated viral entry to Ud agglutinin [33,34]. a lectin predominantly localized in the root of *Urtica dioica*; however, the Ud extract used in the present study did not include root-derived material. While the pseudovirus-based neutralization and Mpro inhibition assays provide valuable mechanistic insights, these findings are derived from surrogate and biochemical assays; therefore, confirmation using replication-competent SARS-CoV-2 infection models is required to establish definitive antiviral efficacy. Moreover, the relatively high concentrations required to achieve these effects may limit direct translational applicability.

Beyond its antiviral activity, UdE displayed a stimulus-dependent immunomodulatory profile rather than uniform cytokine suppression. UdE consistently attenuated IL-1β and IL-6—key mediators of inflammatory tissue damage under virus conditioned and LPS-induced settings. Mechanistic studies have demonstrated that UdEs potently inhibit NF-κB activation by stabilizing IκB and preventing NF-κB nuclear translocation, thereby attenuating transcriptional programs governing pro-inflammatory cytokine expression, including IL-1β and IL-6 while preserving cell viability and tissue integrity [35,36,37]. Within this framework, flavonoids such as quercetin emerge as major contributors to NF-κB inhibition and cytokine attenuation, yet Parente et al. emphasize that whole-extract activity frequently exceeds that of isolated constituents, suggesting additive or synergistic interactions between phenolic acids, flavonoids, and antioxidant components [36]. In contrast, IL-8 regulation was context-specific: UdE increased IL-8 under viral and inflammatory stimulation but reduced basal IL-8 levels in the absence of stimuli, indicating intrinsic anti-inflammatory activity alongside preserved chemokine responsiveness. This pattern is biologically coherent given IL-8’s dual role in neutrophil recruitment and early antiviral defense, particularly during respiratory viral infections, where controlled induction supports host immunity while excessive or sustained responses drive pathology [38,39,40,41]. This dual pattern highlights the context-dependent nature of the immune response and suggests that UdE may modulate, rather than simply suppress, inflammatory signaling. However, the potential for pro-inflammatory effects cannot be excluded, particularly under different exposure conditions or concentrations, and warrants further investigation. Collectively, these findings indicate that UdE functions as an immune-balancing regulator, selectively dampening harmful NF-κB–driven cytokine amplification (IL-1β, IL-6) while maintaining context-appropriate IL-8 signaling, likely through synergistic actions of flavonoids and phenolic constituents rather than broad immunosuppression. Clinical evidence further supported that IL-8 plays a context-dependent role in SARS-CoV-2 infection rather than serving as a uniformly pathological marker. Longitudinal and *in vitro* analyses show that controlled, antigen-induced IL-8 responses contribute to early antiviral immunity, whereas excessive or sustained IL-8 production is linked to impaired neutralizing antibody durability, immune dysregulation, and disease severity [38,41,42].

The present study addresses this gap by providing direct *in vitro* evidence that UdEs exert coordinated antioxidant, immunomodulatory, and antiviral effects. These results underscore the originality of this work and support the potential relevance of UdE as a multi-functional bioactive extract for further preclinical investigation.

## 4. Materials and Methods

### 4.1. Physicochemical Analysis of UdE

*Urtica dioica* (whole plant without root) extracts without any additives was purchased from IMMUNAT Herbal Medicine Company (Muğla, Turkey). They were harvested from the Isparta and Denizli, regions in western Türkiye. They were extracted with CH_3_CH_2_OH:H_2_O (50:50, *v*/*v*) and the extraction yield was 15.54% 0.13 g/100 g dry plant materisl as reported by the manufacturer. The extract was lyophilized at −60 °C and 0.1 mbar using a lyophilizer (CHRIST Alpha 1-2 LDplus, Martin Christ, Osterode am Harz, Germany) and stored at −80 °C until use. All experiments were conducted using a single, well-characterized extract batch to ensure internal consistency, while batch-to-batch variability was not evaluated within the scope of this study.

#### 4.1.1. Microtiter Quantification

Microtiter based quantification of UdE was performed to determine its total phenolic content (TPC), total flavonoid content (TFC), flavanol content, total ortho-diphenolic content (TdOPC), and aucubin content. The results are expressed as gallic acid equivalents (GAE), quercetin equivalents (QE), pyrocatechol equivalents (PE), and aucubin equivalents (AE), respectively, and reported as mg/g of extract. The external standard calibration curves of the quantified TPC, TFC, and TdOPC are presented in the Supplementary Materials (Figures S1–S3). Collectively, these findings demonstrate that UdE possesses a phytochemical profile enriched in polyphenolic and flavonoid compounds, which may contribute to its observed biological activities.

#### 4.1.2. High Performance Liquid Chromatography (HPLC)

The UdE sample in 100 mg in distilled water was sonicated for 15 min and subsequently centrifuged to remove insoluble material. For solid-phase extraction (SPE) with a C18 cartridge rinsed with methanol and equilibrated using acidified water, the extract was loaded to the cartridge and washed with acidified water to eliminate residual impurities and allowed to air-dry for 24 h. The elution of retained analytes was achieved using methanol. Chromatographic analysis was carried out using a Shimadzu LC-20 AB HPLC system (Shimadzu, Kyoto, Japan) equipped with a binary pump, an autosampler (SIL-20AC), and a UV–Vis detector (SPD-20AV) (Shimadzu, Kyoto, Japan). Detection was performed at wavelengths of 280 nm and 320 nm. Compound identification was based on comparison with authentic standards and corresponding relative retention times. Separation was conducted on an ACE^®^ C18 reversed-phase column (250 × 4.6 mm i.d., 5 μm; Advanced Chromatography Technologies Limited, Aberdeen, Scotland) using a 20 μL injection volume. The mobile phase consisted of solvent A (0.2% trifluoroacetic acid in water) and solvent B (methanol/acetonitrile mixture), delivered at a constant flow rate of 1.2 mL/min. Gradient elution was initiated with 95% A and 5% B for 0–30 min, followed by a linear increase to 30% B between 30 and 35 min and to 50% B from 35 to 40 min. The proportion of solvent B was then increased to 100% over 40–50 min. Initial conditions were subsequently restored and maintained for 2 min to ensure column re-equilibration. The column temperature was maintained at 45 °C using a Shimadzu CTO-10AC column oven (Shimadzu, Kyoto, Japan), while the autosampler was set at 4 °C to reduce analyte degradation. For standard preparation and calibration, individual reference compounds purchased from Sigma-Aldrich (St. Louis, MO, USA)—including gallic acid, chlorogenic acid, caffeic acid, vanillic acid, p-coumaric acid, vanillin, ferulic acid, 2-hydroxycinnamic acid, rosmarinic acid, quercetin, apigenin, kaempferol, hesperetin, 5,7-dihydroxyflavone, and rutin—were prepared by dissolving 1 mg of each compound in 10 mL of methanol to obtain stock solutions. Working standards were prepared by diluting 1 mL of each stock solution to 10 mL with methanol. Seven-point calibration curves were generated using concentrations ranging from 0.05 to 5 mg/L. Aliquots (1 mL) of each calibration level were transferred into HPLC vials for analysis. For extract analysis, 1 mL of UdE was directly transferred into an HPLC vial and injected under the same chromatographic conditions.

### 4.2. Antioxidant Potential of UdE

#### 4.2.1. Chemical-Based Antioxidant Assays

Cupric Reducing Antioxidant Power Assay: This assay employs the use of copper (II) neocuproine reagent as the chromogenic oxidizing agent. This assay is based on reduction of Cu(II)-neocuproine complex to highly colored Cu(I)-neocuproine complex, which was measured at 450 nm absorbance. The reducing capacity of the extracts was determined using cupric ion reducing antioxidant capacity by [43]. A standard calibration curve was made with gallic acid (CAS No: 149-91-7, Sigma Aldrich) with an R2 value of 0.97. For this assay, 20 μL of UdE at a dilution factor of 100 was added with 100 μL of 10 mM CuCl_2_ solution followed by 100 μL of 1 M ammonium acetate buffer at pH 7.0. To the resulting solution, 100 μL of 7.5 mM neocuproine ethanolic solution was added and the reaction was allowed to proceed for 30 min after which the absorbance at 405 nm was recorded. Results were expressed and calculated from an ascorbic acid calibration curve and expressed as mg/mL ascorbic acid (CAS No: 50-81-7, Sigma-Aldrich).

Ferric Reducing Antioxidant Power Assay (FRAP): It is based on the reduction of the ferric tripyridyltriazine (Fe(III)(TPTZ)2) complex to ferrous form under acidic conditions, which produces an intense blue coloration. The assay was performed according to the spectrophotometric method described by Lia and Baron (2025) [43]. A calibration curve was generated using ascorbic acid as the reference standard, showing excellent linearity (R^2^ = 1.0). The FRAP working reagent was freshly prepared by combining 300 mM acetate buffer, 10 mM TPTZ dissolved in 400 mM HCl, and 20 mM FeCl_3_ in a volumetric ratio of 10:1:1. For analysis, 10 μL of UdE diluted 1:100 was mixed with 200 μL of the FRAP rea-gent and vortexed thoroughly. In the presence of antioxidant compounds, Fe^3+^–TPTZ is reduced to Fe^2+^–TPTZ, resulting in a measurable increase in absorbance at 593 nm. Anti-oxidant capacity was quantified using the ascorbic acid standard curve and expressed as mg/mL ascorbic acid equivalents.

DPPH (2,2-Diphenyl-1-picrylhydrazyl) Radical Scavenging Assay: The free radical scavenging capacity of UdE was evaluated using the DPPH (2,2-diphenyl-1-picrylhydrazyl) assay, which employs a stable nitrogen-centered radical. A fresh 60 μM DPPH solution was prepared daily in methanol and protected from light at 4 °C until use. For analysis, 50 μL of the phenolic-enriched stock solution was dispensed into a 96-well microplate and serially diluted two-fold to obtain a concentration range. Control wells containing 50 μL of methanol served as DPPH blanks. Subsequently, 150 μL of the DPPH solution was added to each well, and the reaction mixtures were incubated for 30 min in the dark at room temperature. Absorbance was measured at 560 nm using a micro-plate reader. The scavenging activity of UdE was expressed as the effective concentration required to quench 50% of DPPH radicals (EC_50_).

ABTS (2,2′-Azino-bis(3-ethylbenzothiazoline-6-sulfonic Acid) Radical Cation Scavenging Assay: The antioxidant capacity of UdE was evaluated using the ABTS [2,2′-azinobis(3-ethylbenzothiazoline-6-sulfonic acid)] radical cation decolorization assay, which offers greater methodological flexibility than the DPPH assay and allows assess-ment under varying pH conditions. The procedure was carried out according to the meth-od described Lia and Baron (2025) [43]. The ABTS•^+^ radical was generated by re-acting a 7 mM ABTS stock solution with 2.45 mM potassium persulfate, followed by incu-bation of the mixture in the dark at room temperature for 12 h. Prior to analysis, the result-ing blue-green ABTS•^+^ solution was diluted with methanol to obtain an absorbance of 0.70 at 734 nm. For the assay, 50 μL of the concentrated extract was added to a 96-well micro-plate and serially diluted two-fold to achieve a range of concentrations. Control wells con-taining 50 μL of methanol were included as negative controls. Subsequently, 280 μL of the ABTS•^+^ working solution was dispensed into each well, and the reaction mixtures were incubated for 5 min at 30 °C. Absorbance was recorded at 450 nm using a microplate reader. ABTS radical scavenging activity was expressed as percentage inhibition, calculated using the following equation:

% *ABTS inhibition* = 100 − 100 (*ABssamble*)/(*ABscontrol*)

Hydrogen Peroxide Scavenging Assay: H_2_O_2_ scavenging capacity of UdE was evaluated using an assay based on the formation of a colored complex between ferrous ions (Fe^2+^) and 1,10-phenanthroline. The procedure was performed with minor modifications to the method described by Lia and Baron (2025) [43]. Quantification was achieved using a gallic acid standard curve, which demonstrated excellent linearity (R^2^ = 1.00). Briefly, 25 μL of 1 mM iron(II) sulfate solution was added to each well of a 96-well microplate, followed by the addition of 150 μL of UdE diluted 1:100. After vigorous mixing, 63 μL of 50 mM hy-drogen peroxide was introduced to initiate the reaction. Following a 5 min incubation, 150 μL of 1 mM 1,10-phenanthroline was added to form the Fe^2+^–phenanthroline complex. The mixture was incubated for an additional 10 min, and absorbance was measured at 490 nm using a microplate reader.

#### 4.2.2. Cell-Based Antioxidant Assays

The cytotoxic potential of UdE was evaluated using a panel of cell lines commonly employed in SARS-CoV-2 research due to their high ACE2 expression [44,45]. These included African green monkey kidney cells (Vero E6, CRL-1586), human embryonic kidney cells (HEK293T, CRL-11268), human lung adenocarcinoma cells (Calu-3, HTB-55), and human colon adenocarcinoma cells (Caco-2, HTB-37) were maintained in Dulbecco’s Modified Eagle Medium (DMEM; Gibco (Grand Island, NY, USA), p#41965) supplemented with 10% fetal bovine serum (FBS; Gibco, p#10500) and 1% antibiotic–antimycotic solution. Cells were incubated at 37 °C in a humidified atmosphere containing 5% CO_2_ and sub-cultured upon reaching 70–80% confluency. All cell-based assays were performed using at least three independent biological replicates (*n* ≥ 3), each measured in six technical replicates.

Assessment of Cytotoxicity: Vero E6 (p# 40–50), HEK293T (p# 22–25), Calu-3 (p# 55–60), and Caco-2 (p# 50–65) cells were seeded into 96-well plates at densities of 7 × 10^3^, 5 × 10^3^, 15 × 10^3^, and 15 × 10^3^ cells per well, respectively, and allowed to adhere for 24 h. The culture medium was then replaced with fresh DMEM containing UdE at concentrations ranging from 78.1 to 5000 µg/mL, followed by incubation for 24, 48, or 72 h. Briefly, MTT reagent (0.5 mg/mL in DMEM; Sigma-Aldrich, #M5655) was added to each well and incubated for 4 h at 37 °C. The resulting formazan crystals were solubilized with dimethyl sulfoxide (DMSO; Sigma-Aldrich, #D8418), and plates were agitated for 2 h to ensure complete dissolution. Absorbance was measured at 570–630 nm using a microplate reader (BioTek Instruments, Winooski, VT, USA). Cell viability was calculated by normalizing absorbance values of treated samples to untreated controls and expressed as a percentage of control cells.

Intracellular ROS Measurement (DCFDA Assay): Reactive oxygen species (ROS) scavenging ability of UdE was investigated by performing fluorescence 2′,7′-dichlorofluorescein diacetate (DCFDA, #D6883 Sigma-Aldrich) assay measured intracellular ROS generation and oxidative stress levels. Vero E6, HEK293T, Caco-2 and Calu-3 cells were cultured with the same cell density as described above and then incubated at 37 °C and 5% CO_2_ for 24 h. Subsequently, UdE at less than TD30 concentration ranged from 50 to 750 µg/mL were applied to the tested cell monolayers and 10 mM N-acetyl cysteine (NAC) (#A7250 Sigma-Aldrich Chemical Co.) used as a positive control then the plates were incubated at 37 °C with 5% CO_2_ for 24 h. In parallel, cell viability of the tested cells was evaluated at the same conditions by MTT assay. After that, the cell monolayers were washed twice with PBS and 10 μM DCFDA was added then incubated for 30 min at 37 °C. 100 μM hydrogen peroxide (H_2_O_2_, #H1009 Sigma-Aldrich Chemical Co.) was added to induce the oxidative stress. The fluorescence was measured at Ex/Em = ~485/~535 nm using a microplate reader (Cytation 7, Biotek, USA) during 60 min.

RT-qPCR Analysis: HEK293T cells were seeded into 6-well plates at a density of 2.5 × 10^5^ cells/well and incubated at 37 °C, 5% CO_2_ for 24 h. Following incubation, the cells were treated with 750 µg/mL UdE for 24 h. Oxidative stress was subsequently induced by treatment with 100 µM H_2_O_2_, and the cells were incubated for 8 h and 12 h. NAC (10 mM) was used as a positive antioxidant control. Total RNA was isolated using the Zymo Quick-RNA MiniPrep Plus Kit (#R1057, Zymo Research, Irvine, CA, USA) according to the manufacturer’s instructions. RNA concentration and purity were determined using a NanoDrop spectrophotometer (#13-400-518, Thermo Fisher Scientific, Waltham, MA, USA). For cDNA synthesis, 1000 ng of total RNA was reverse transcribed using the iScript cDNA Synthesis Kit (#170-8891, Bio-Rad, Hercules, CA, USA) following the manufacturer’s protocol. Quantitative RT-PCR was performed using the iTaq Universal SYBR Green Supermix (#172-5124, Bio-Rad, USA) on a Bio-Rad CFX Opus 96 (#CFX Opus 96, Bio-Rad, USA). Each reaction was carried out in a final volume of 20 µL containing SYBR Green master mix, cDNA template, and gene-specific primers. Primers were used at a final concentration of 10 µM with 0.5 µL of forward and 0.5 µL of reverse primers added per reaction. All primers were synthesized by Sentebiolab (Ankara, Turkey). The thermal cycling conditions were as follows: initial denaturation at 94 °C for 10 min, followed by 40 cycles of denaturation at 94 °C for 5 s and annealing/extension at 60 °C for 30 s. A melting curve analysis (94 °C for 10 s, 65 °C for 60 s, and 97 °C for 10 s) was performed to confirm amplification specificity [46]. The primer sequences used were in this study (5′–3′): *Nrf2* forward: TTCCCGGTCACATCGAGAG, *Nrf2* reverse: TCCTGTTGCATACCGTCTAAATC; *HO-1* forward: GGCAGAGGGTGATAGAAGAGG, *HO-1* reverse: TAAGGACCCATCGGAGAAGC; *GAPDH* forward: ATGAAGGGGTCATTGATGG, GAPDH reverse: AAGGTGAAGGTCCGAGTCAA.

#### 4.2.3. Morphological and Apoptotic Features of the Cell Lines upon Exposure to UdE

The assessment of cellular morphology and apoptosis was performed to evaluate the cytotoxic and cell death-associated effects induced by UdE exposure at the cellular level. Hematoxylin and eosin (H&E) staining was employed to assess cellular morphology, including nuclear, cytoplasmic, and extracellular matrix features. Hematoxylin selectively binds to negatively charged nuclear components, resulting in blue–purple nuclear staining, whereas eosin interacts with cytoplasmic and extracellular proteins to produce pink to red coloration. Vero E6, HEK293T, Calu-3, and Caco-2 cells were seeded in 24-well plates at a density of 1–2 × 10^4^ cells per well and incubated at 37 °C under 5% CO_2_ for 24 h. Subsequently, the cell monolayers were treated with UdE at concentrations ranged from 1250 to 3500 µg/mL followed by an additional 24 h incubation. After treatment, the cells fixed with 4% paraformaldehyde for 15 min at RT and permeabilized with 0.2% Triton X-100 for 10 min, Hematoxylin solution (Bio-Optica, Milan, Italy) was applied for 1 min, followed by washing under running tap water. Then the cells were counterstained with eosin (Bio-Optica, Milano, Italy) for 5 min and dehydrated twice with absolute ethanol. The cells were mounted using a mounting medium and the representative images were acquired at 20× magnification using a Cytation™ 7 imaging system (BioTek, USA).

Annexin V-FITC conjugated protein binds to cell surfaces expressing phosphatidylserine, an early apoptosis marker including changes in nuclear morphology, DNA fragmentation and membrane leaflet symmetry. The cells stained with propidium iodide (PI), a non-cell-permeable DNA dye, indicate necrotic cells. The cell monolayers were stained with both annexin V-FITC and PI demonstrate later stage apoptosis and early necrosis [47]. HEK293T and Calu-3 cell lines were seeded at a cell density of 4–8 × 10^4^ cells/cover slips in the 24-well plate and incubated at 37 °C, and 5% CO_2_ for 24 h. The following day, the extract was applied at concentrations ranged from 1500 to 3000 µg/mL and the cells were incubated for 24 h. After washing twice with PBS, Annexin V (#11828681001, Roche, Basel, Switzerland) diluted with Annexin Binding Buffer (20 µg/mL), 1 µg/mL Propodium iodide (#81845, Sigma), 1 µg/mL Hoechts (#565877, Pharmingen, San Diego, CA, USA) was added to each well. The slides were washed with PBS and then the images were taken with 20× magnification using the Cytation 7.

### 4.3. Antiviral Potential of UdE

#### 4.3.1. RBD–ACE2 Binding Inhibitory Potential of UdE

To evaluate the targeted antiviral activity of UdE, inhibition of the SARS-CoV-2 RBD–ACE2 interaction was assessed using a commercial ELISA assay (BPS Bioscience (San Diego, CA, USA), #79931), following the manufacturer’s protocol with minor adaptations. Briefly, 96-well plates pre-coated with recombinant Spike S1 RBD (1 μg/mL in PBS) were incubated overnight at 4 °C. After washing three times with assay buffer, the wells were blocked with 100 µL of blocking solution for 1 h at RT. The plates were then washed again, and 20 µL of assay buffer was added to each well. Spike S1 Neutralizing Antibody (SARS-CoV-2) (Clone: 414-1) (#100793), ACE2-His, PBS and assay buffer were used an internal control, as a positive control and as negative control, respectively. UdE was prepared at the desired final concentrations and added to the wells at a volume of 10 µL per well and UdE dilutions used as interference w/o adding ACE2 and Spike S1 RBD. To ensure solvent consistency, 1x assay buffer containing 5% DMSO was used for both positive and blank control wells. Following a 1 h incubation at RT with gentle shaking, His-tagged ACE2 protein (2.5 ng/µL in assay buffer) was added to each well and incubated for an additional 1 h. The plates were subsequently washed three times and incubated with an HRP-conjugated anti-His antibody for 1 h at RT under gentle agitation. After the final washing steps, chemiluminescent HRP substrate was applied, and luminescence signals were measured using a Cytation™ 7 microplate reader. Inhibition of RBD–ACE2 binding was calculated as a percentage relative to the positive control provided in the assay kit.

#### 4.3.2. Inhibitory Potential of UdE Against SARS-CoV-2 Main Protease Activity

The SARS-CoV-2 Mpro an attractive target for antiviral drug development. The inhibitory activity of UdE derived from the Mediterranean region against SARS-CoV-2 Mpro was evaluated using a commercial fluorometric Mpro Protease Assay Kit with MBP-tagged enzyme (BPS Bioscience, #79955), following the manufacturer’s instructions. Briefly, 30 µL of recombinant Mpro protease (3–5 ng/µL) prepared in assay buffer containing 1 mM dithiothreitol (DTT) was added to the designated wells. Assay buffer containing 1 mM DTT alone served as the blank control, while GC376 (500 µM) was used as the positive inhibitor control and Mpro protease was used as positive control. UdE, prepared in assay buffer containing 1% DMSO, was added to the 96-wells of the black plate and preincubated with the enzyme for 30 min at RT under gentle agitation. UdE tested dilutions was used as interference w/o adding fluorescence agent. Protease activity was then quantified by measuring fluorescence intensity at excitation/emission wavelengths of 360/460 nm using a microplate reader. The percentage inhibition of Mpro activity by UdE was calculated relative to the positive inhibitor control (GC376), which was defined as 100% inhibition.

#### 4.3.3. Antiviral Activity of UdE Against SARS-CoV-2 S1 Pseudovirus Neutralization Assay

The pseudovirus involving SARS-CoV-2 Spike 1 (S1) was prepared as described by Grehan et al. (2015) [48]. A lentiviral pseudovirus system expressing the SARS-CoV-2 spike S1 protein was generated as previously described with minor modifications. Plasmids encoding pCEP4-myc-ACE2 (Addgene #141185), TMPRSS2 (Addgene #53887), spike-18aa (Addgene #149541), psPAX2 (Addgene #12260), and lenti RRL_GFP (Addgene #12252) were isolated using the ZymoPURE™ II Plasmid MidiPrep Kit (Zymo Research, USA) according to the manufacturer’s instructions. Briefly, plasmid constructs in 50 ng were transformed into NEB^®®^ Stable competent Escherichia coli cells (New England Biolabs, Ipswich, MA, USA) by heat shock. Transformed bacteria were recovered in SOC medium at 37 °C with shaking at 225 rpm for 1 h, plated onto LB agar plates, and incubated overnight. Purified plasmids were quantified using a NanoDrop spectrophotometer and subsequently used for transfection experiments in HEK293T cells.

For pseudovirus production and ACE2 expression, HEK293T cells were seeded at a density of 5 × 10^5^ cells per well in 6-well plates and transfected at 70–80% confluence using FuGENE^®^6 transfection reagent (Promega, Madison, WI, USA). For SARS-CoV-2 S1 pseudovirus generation, plasmid mixtures containing 7500 ng lenti RRL_GFP reporter plasmid, 6750 ng psPAX2 packaging plasmid, and 750 ng spike-18aa expression plasmid were combined with FuGENE^®^6 in serum- and antibiotic-free DMEM. For ACE2 expression, 1250 ng each of ACE2 and TMPRSS2 plasmids were similarly prepared. HEK293T cells were co-transfected with ACE2 and TMPRSS2 expression plasmids to mimic physiological SARS-CoV-2 entry conditions, since TMPRSS2 promotes proteolytic activation of the spike protein, thereby enhancing ACE2-dependent viral entry. After incubation for 25–30 min at room temperature, the plasmid–reagent complexes were added dropwise to the cells. Following 14–16 h of transfection, the medium was replaced with DMEM supplemented with 10% fetal bovine serum and 1% penicillin/streptomycin. Supernatants containing pseudovirus particles were harvested after 72 h, clarified by filtration through a 0.45 µm membrane filter, and stored at −80 °C until use.

For pseudovirus entry assays, HEK293T cells expressing human ACE2 were seeded at a density of 2 × 10^4^ cells per well in black 96-well plates and incubated for 24 h at 37 °C in a humidified atmosphere containing 5% CO_2_. To assess the inhibitory effect of UdE on viral entry, pseudovirus S1 particles were preincubated with UdE for 1 h at 37 °C prior to addition to the target cells. The cell monolayers were then incubated for 72 h under standard culture conditions. Pseudovirus infection efficiency was quantified by measuring GFP fluorescence intensity using a microplate reader. In parallel, cell viability was evaluated in the same wells using the CellTiter-Glo^®^ Luminescent Cell Viability Assay (Promega, #G7571). Neutralization efficiency was calculated relative to fluorescence signals obtained from conditioned media collected from mock-transfected control cells. In addition, pseudovirus entry data were adjusted to the cell viability to minimize the contribution of cytotoxic effects, however, residual effects of sub-cytotoxic stress cannot be fully excluded. This experiment was performed using at least three independent biological replicates (*n* ≥ 3), each measured in six technical replicates.

### 4.4. Immunomodulatory Effects of UdE upon SARS-CoV-2 Infection

To assess the immunomodulatory effects of UdE, co-culture was established using ACE2- transfected HEK293T cells and macrophage differentiated human monocytic THP-1 cells (ATCC^®^ TIB-202) which was specifically designed to mimic epithelial–immune cell communication during SARS-CoV-2 infection. This system allows the exchange of soluble factors between the two cell types while preventing direct cell–cell contact. THP-1 monocytes were seeded at a density of 2 × 10^5^ cells per well in 24-well transwell plates and differentiated into macrophages by treatment with 10 nM phorbol 12-myristate 13-acetate (PMA; CAS No:16561-29-8, Sigma-Aldrich) for 24 h. Following differentiation, PMA-containing medium was replaced with fresh complete medium, and the cells were incubated for an additional 24 h to allow recovery and maturation. HEK293T cells was transfected with ACE2 and TMPRSS2 plasmids as described in PRNT assay previously and ACE2- expressing HEK293T cells were seeded into the transwell inserts at a density of 2 × 10^4^ cells per well and co-cultured with previously seeded THP-1 derived macrophages on the basolateral side of the insert for 24 h at 37 °C in a humidified atmosphere containing 5% CO_2_. UdE (200 µg/mL) was then added to the co-culture system and incubated for 2 h prior to exposure to SARS-CoV-2 spike S1 pseudovirus particles. The cultures were subsequently maintained for 72 h under standard incubation conditions. This experiment was performed using at least three independent biological replicates (*n* ≥ 3), each measured in technical triplicates. Lipopolysaccharide (LPS; 0.5 µg/mL; Sigma-Aldrich) was used as a positive control for cytokine induction. After the exposure period, culture supernatants were collected from both the inserts and the wells, gradually cooled, and stored at −80 °C until further analysis. Therefore, the observed cytokine modulation reflects the integrated response of the co-culture system, rather than being attributable exclusively to either direct effects on THP-1 macrophages or indirect effects mediated through HEK293T cells.

Quantification of Cytokine Levels by ELISA: The concentrations of tumor necrosis factor-alpha TNF-α (#430204), IL-1β (#437004), IL-6 (#430504) and IL-8 (#431504) in the collected supernatants were quantified using Human ELISA MAX™ Deluxe Set kits (BioLegend, San Diego, CA, USA) according to the manufacturer’s instructions. Briefly, high-binding 96-well microplates (Nunc™ MaxiSorp™, Thermo Fisher Scientific) were coated overnight at 4 °C with capture antibodies diluted in 1x assay diluent. After washing with PBS containing 0.05% Tween-20 (PBST), the plates were blocked for 1 h at RT using the appropriate blocking solutions specified for each cytokine. Standards and appropriately diluted samples (100 µL per well) were added and incubated for 2 h at RT. Following washing steps, biotinylated detection antibodies were applied for 1 h, after which avidin–horseradish peroxidase conjugate was added. Color development was achieved using the kit specific tetramethylbenzidine (TMB) based substrates, and reactions were terminated with 2 N sulfuric acid. Absorbance was measured at 450 nm with wavelength correction at 570 nm using a microplate reader. Cytokine concentrations were calculated from standard curves and expressed in pg/mL. All experiments were performed in triplicate to ensure reproducibility and reliability of the results.

### 4.5. Statistical Analysis

Statistical analyses were conducted using GraphPad Prism version 8.0.2. Differences between groups were evaluated by one-way or two-way analysis of variance (ANOVA) followed by Tukey’s multiple comparisons. To control the possible false-positive findings arising from multiple comparisons, post hoc analysis was performed using Tukey’s multiple comparisons test following ANOVA. All experiments were performed using at least three independent biological replicates (*n* ≥ 3), each measured in technical triplicates unless otherwise stated. Data are presented as mean ± standard deviation (SD) from three independent experiments (*n* = 3), and *p* values are reported where applicable, and values of *p* < 0.05 were considered statistically significant.

## 5. Conclusions

In conclusion, the present study provides comprehensive *in vitro* evidence that *Urtica dioica* extract (UdE) exerts coordinated antioxidant, antiviral, and immunomodulatory activities relevant to SARS-CoV-2 infection. Moving beyond predominantly *in silico* predictions, our findings experimentally demonstrate that UdE functions as a multi-target bioactive extract acting at distinct yet mechanistically interconnected levels of viral pathogenesis and host response.

UdE preserved cellular viability and morphology while promoting a balanced redox state under oxidative stress conditions, supporting a regulatory—rather than indiscriminate—antioxidant profile. These findings indicate that UdE functions as a modulator of redox homeostasis via activation of the Nrf2/HO-1 pathway, which may also contribute to its potential antiviral effects. By attenuating excessive ROS without abolishing physiologically relevant redox signaling, UdE may help maintain host antiviral competence while limiting oxidative tissue damage. This redox-modulating capacity is consistent with its phenolic-rich composition, particularly chlorogenic acid and quercetin, and appears reproducible across different cell types.

At the antiviral level, UdE demonstrated potent inhibition of SARS-CoV-2 Mpro with a low micromolar–equivalent EC_50_, supported by both biochemical screening and structure-based docking analyses. In parallel, UdE markedly disrupted RBD–ACE2 binding and partially inhibited spike-pseudotyped viral entry, reflecting mechanistic interference with early stages of infection. The observed differences between biochemical binding assays and pseudovirus systems are consistent with known assay-specific dynamics and do not diminish the biological relevance of the entry-modulating activity. Importantly, these antiviral effects are attributable primarily to chlorogenic acid, with additive contributions from quercetin and other phytochemicals within the extract matrix.

Beyond direct antiviral actions, UdE displayed a stimulus-dependent immunomodulatory profile characterized by attenuation of NF-κB–associated cytokines (IL-1β, IL-6) while preserving context-appropriate IL-8 responses. This selective immune balancing—rather than broad immunosuppression—suggests that UdE may help limit inflammatory amplification without compromising early antiviral defense.

Collectively, these findings position UdE as a biologically active, multi-functional plant-derived extract that integrates redox regulation, viral enzyme inhibition, entry interference, and immune modulation. The originality of this work lies in experimentally validating these complementary mechanisms within a unified framework, thereby strengthening the translational potential of UdE as a supportive modulatory approach in viral infections.

## Figures and Tables

**Table 1:** TD_30_ and TD_50_ values of UdE upon applied to Vero E6, HEK293T, Caco-2 and Calu-3 cell lines for 24 h, 48 h, and 72 h.

Exposure Time (h)	Vero E6	HEK293T	Caco-2	Calu-3
TD_30_ Values (μg/mL)				
24	2397.75 ± 769.70	1023.91 ± 76.60	2869.58 ± 467.72	3313.69 ±154.52
48	1677.55 ± 606.45	574.15 ± 9.54	2735.92 ± 339.98	2435.00 ± 478.84
72	738.51 ± 210.86	730.03 ± 15.91	1962.40 ± 113.97	1166.43 ± 179.06
TD_50_ Value (μg/mL)				
24	3348.39 ± 618.87	1680.07 ± 127.24	3660.41 ± 293.04	4001.54 ± 83.41
48	2789.87 ± 641.56	1125.04 ± 75.98	3475.42 ± 315.87	3480.43± 449.54
72	1274.38 ± 275.47	1197.22 ± 35.58	2913.62 ± 104.15	1932.63 ± 307.82

## Data Availability

The original contributions presented in this study are included in the article/Supplementary Materials. Further inquiries can be directed to the corresponding author.

## Supplementary Materials

The following supporting information can be downloaded at: https://www.mdpi.com/article/10.3390/ph19050693/s1, Figure S1: Calibration curve for the determination of total phenolic content (TPC); Figure S2: Calibration curve for the determination of total flavonoid content (TFC); Figure S3: Calibration curve for the determination of total orthodiphenolic content (TdOPC); Table S1: External standard calibration curves of the quantified phenolic acids and flavonoids; Figure S4a,b: Calibration curves, LOD and LOQ for the selected standards ((1) gallic acid, (2) chlorogenic acid, (3) caffeic acid (4) vanillic acid, (5) p-coumaric acid, (6) vanillin, (7) ferulic acid, (8) 2-hydroxyccynnamic acid (9) rosmarinic acid, (10) hesperidin, (11) quercetin, (12) apigenin, (13) kaempferol, (14) hesperetin, (15) chrysin, (16) rutin) with various ethanol concentrations for HPLC-UV analysis of the phenolic compounds in Ud extracts; Figure S5. Example of chromatograms with variation in ethanol concentration: (a) full-scale and (b) zoomed-in HPLC-UV chromatograms of the phenolic compounds in UACPE extracts with various ethanol concentrations. Annotations: (1) gallic acid, (2) chlorogenic acid, (3) caffeic acid (4) vanillic acid, (5) p-coumaric acid, (6) vanillin, (7) ferulic acid, (8) 2-hydroxyccynnamic acid, (9) rosmarinic acid, (10) hesperidin, (11) quercetin, (12) apigenin, (13) kaempferol, (14) hesperetin, (15) chrysin, (16) rutin; Figure S6: HPLC chromatographic profiles of UdE at 280 nm (A) and 320 nm (B). Peaks corresponding to key phenolic constituents are annotated: gallic acid (GA; tR = 3 min; LOD = 0.266 µg/mL; LOQ = 0.80 µg/mL), chlorogenic acid (CGA; tR = 12 min; LOD = 0.505 µg/mL; LOQ = 1.53 µg/mL), quercetin (Que; tR = 97 min; LOD = 0.586 µg/mL; LOQ = 1.77 µg/mL) and caffeic acid (CA; tR = 88 min; LOD = 0.600 µg/mL; LOQ = 1.82 µg/mL). Retention times (tR) are reported in minutes; LOD and LOQ values are reported as calculated for the method. Refs. [49,50,51,52,53] cited in Supplementary Materials.

## Abbreviations

The following abbreviations are used in this manuscript:

ABTS	2,2′-azino-bis(3-ethylbenzothiazoline-6-sulfonic acid)
ACE2	angiotensin-converting enzyme 2
Annexin V-FITC	fluorescein isothiocyanate–conjugated annexin V
CUPRAC	cupric reducing antioxidant capacity
DCFDA	2′,7′-dichlorodihydrofluorescein diacetate
DMEM	Dulbecco’s modified Eagle’s medium
DMSO	dimethyl sulfoxide
DPPH	2,2-diphenyl-1-picrylhydrazyl
DTT	dithiothreitol
ELISA	enzyme-linked immunosorbent assay
FRAP	ferric reducing antioxidant power
H&E	hematoxylin and eosin
HPLC	high-performance liquid chromatography
IC30	30% inhibitory concentration
IC50	half maximal inhibitory concentration
IL-1β	interleukin 1 beta
IL-6	interleukin 6
IL-8	interleukin 8
Mpro	main protease
MTT	3-(4,5-dimethylthiazol-2-yl)-2,5-diphenyltetrazolium bromide
NAC	N-acetyl-L-cysteine
NF-κB	nuclear factor kappa B
PI	propidium iodide
RBD	receptor-binding domain
ROS	reactive oxygen species
SARS-CoV-2	severe acute respiratory syndrome coronavirus 2
SPE	solid-phase extraction
TdPC	total ortho-diphenolic content
TFC	total flavonoid content
TNF-α	tumor necrosis factor alpha
TPC	total phenolic content
UdE	*Urtica dioica* extract
