# Supplementary material for: Integrated Evaluation of Urtica dioica Extract Assessing Physiochemical Analysis with Antioxidant, Antiviral, and Immunomodulatory Effects Against SARS-CoV-2
Source: Pharmaceuticals (Basel). 2026 Apr 28;19(5):693. doi: 10.3390/ph19050693 (PMC13209815; doi:10.3390/ph19050693)
Supplement: Supplementary file 1 [file pharmaceuticals-19-00693-s001.zip › pharmaceuticals-4179782-supplementary.pdf]

SUPPLEMENTARY MATERIALS

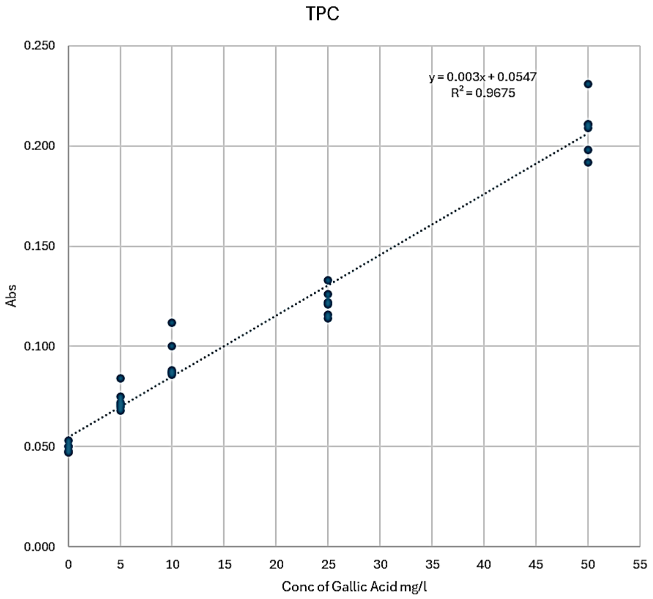

Figure S1. Calibration Curve for the determination of Total Phenolic Content (TPC).

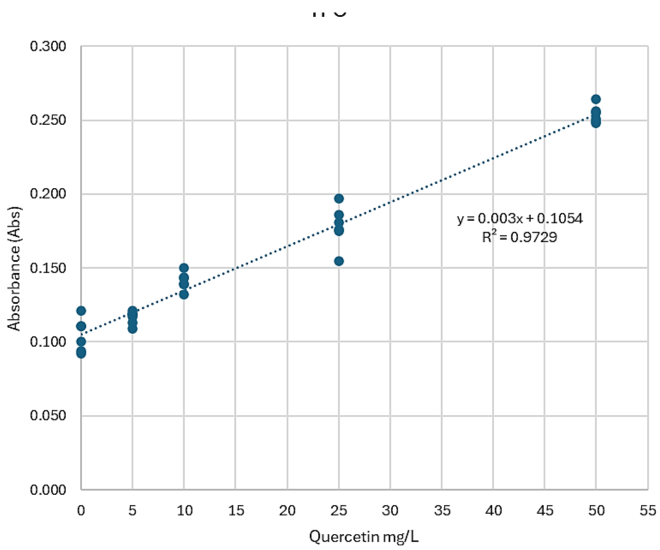

Figure S2. Calibration Curve for the determination of Total Flavonoid Content (TFC).

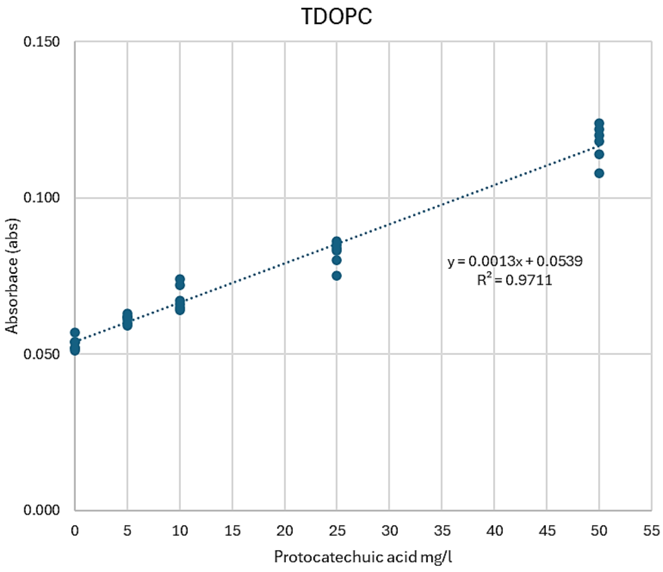

Figure S3. Calibration Curve for the determination of Total Orthodiphenolic Content (TdOPC).

The selection of reference standards for HPLC analysis based on previous studies was reported that UdE is rich in phenolic acids and flavonoids, particularly chlorogenic, caffeic, ferulic, p-coumaric acids, gallic and vanillic acids [1-4], as well as, flavonoids such as quercetin, rutin, and kaempferol [5]. Based on this well-established phytochemical profile, the selected standards were chosen to represent the major compound classes present in UdE.

All reference compounds used for HPLC analysis of UdE were commercially purchased from Sigma-Aldrich, and their corresponding catalog numbers and purity/assay values (≥94–99%) are explicitly reported. In addition, the table includes full analytical validation parameters for each standard, including calibration curve data (slope, intercept, standard error), limits of detection (LOD), limits of quantification (LOQ), linearity ($R^2 > 0.993$), and accuracy (%), confirming the reliability and robustness of the method. No compounds were isolated in-house, and all standards were used as received according to the manufacturer's specifications.

Table S1. External standard calibration curve of the quantified phenolic acids and flavonoids

Standard	Sigma-Aldrich Catalog No.	Purity / Assay	Slope	Intercept	Standard Error	LOD (mg/L)	LOQ (mg/L)	R^2	Accuracy %
**Gallic Acid**	27645	≥99% (HPLC)	13181	63	832	0.266	0.807	0.999	98.06
**Chlorogenic Acid**	C3878	≥95% (titration)	6680	-889	704	0.505	1.531	0.998	97.59
**Caffeic Acid**	C0625	≥98%	14749	505	1848	0.600	1.819	0.998	104.55
**Vanillic Acid**	94770	≥97%	11718	345	919	0.376	1.139	0.999	101.91
**p-Coumaric Acid**	C9008	≥98%	28140	1667	692	0.344	1.043	0.999	99.89
**Vanillin**	V1104	≥99%	23701	141	763	0.450	1.365	0.998	101.40
**Ferulic Acid**	128708	≥99%	17112	2153	1207	0.988	2.993	0.993	104.54
**Hydroxy-cinnamic acid**	H22809	≥98%	36314	-3242	1084	0.452	1.368	0.998	102.16
**Rosmarinic Acid**	R4033	≥98%	12331	-1814	529	0.601	1.820	0.997	98.45
**Quercetin**	Q4951	≥95%	10757	-2965	493	0.586	1.776	0.998	102.66
**Apigenin**	A3145	≥97%	14667	-1621	656	0.572	1.733	0.998	99.46
**Kampherol**	60010	≥97%	9656	-1047	356	0.471	1.428	0.999	101.87
**Hesperitin**	H5254	≥95%	15475	-1047	665	0.423	1.112	0.998	99.69
**5,7Dihydroxy-flavone**	C80105	≥97%	12818	1916	365	0.399	1.208	0.999	99.21
**Rutin**	R5143	≥94%	30296	870	751	0.375	1.135	0.999	99.46

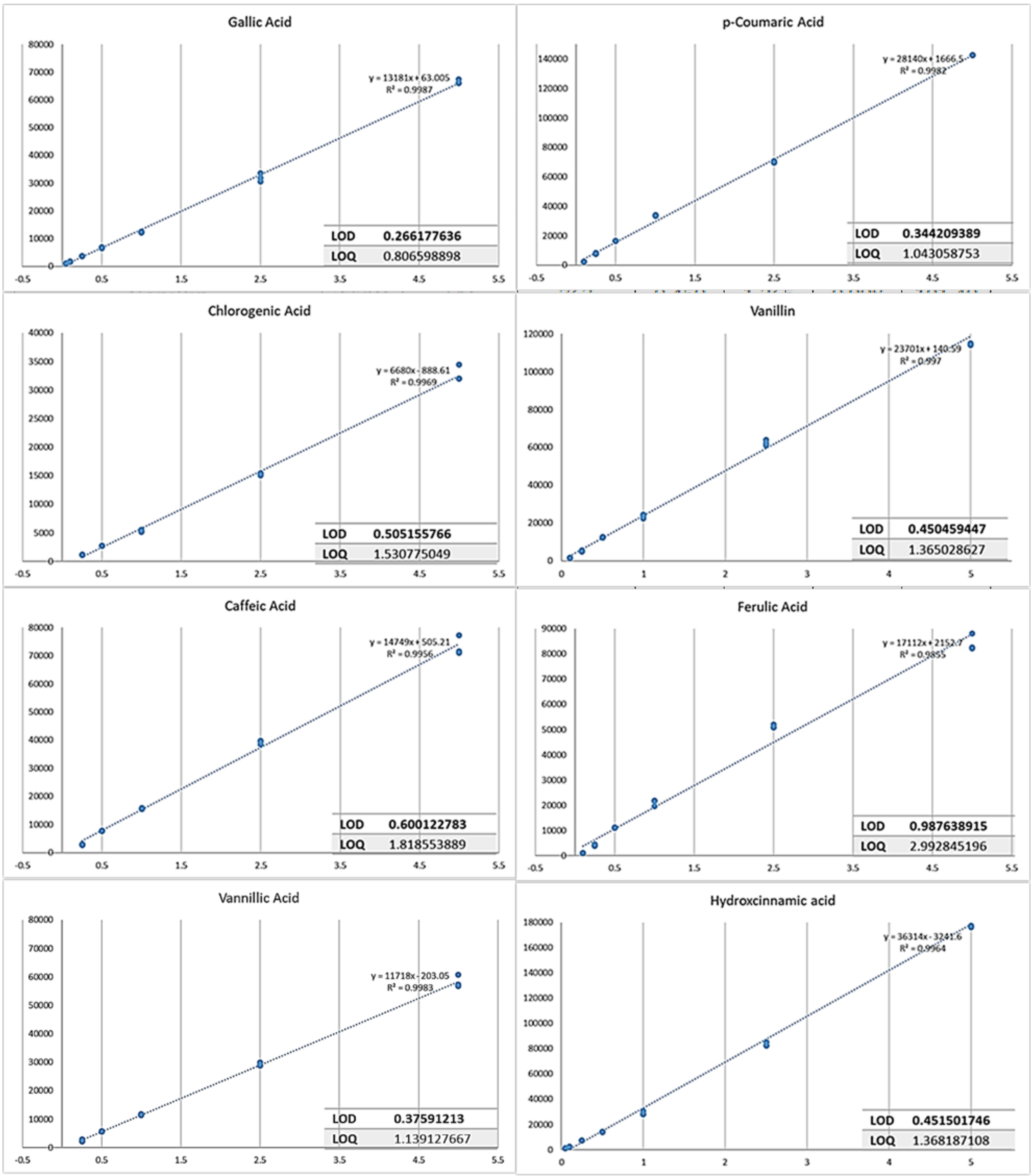

Figure S4a. Calibration Curves, LOD and LOQ for the selected Standards ((1) Gallic acid, (2) Chlorogenic acid, (3) Caffeic acid (4) Vanillicacid, (5) p-Coumaric acid, (6) Vanillin, (7) Ferulic acid, (8) 2-hydroxyccynnamic acid) with various ethanol concentrations for HPLC-UV analysis of the phenolic compounds in Ud extracts.

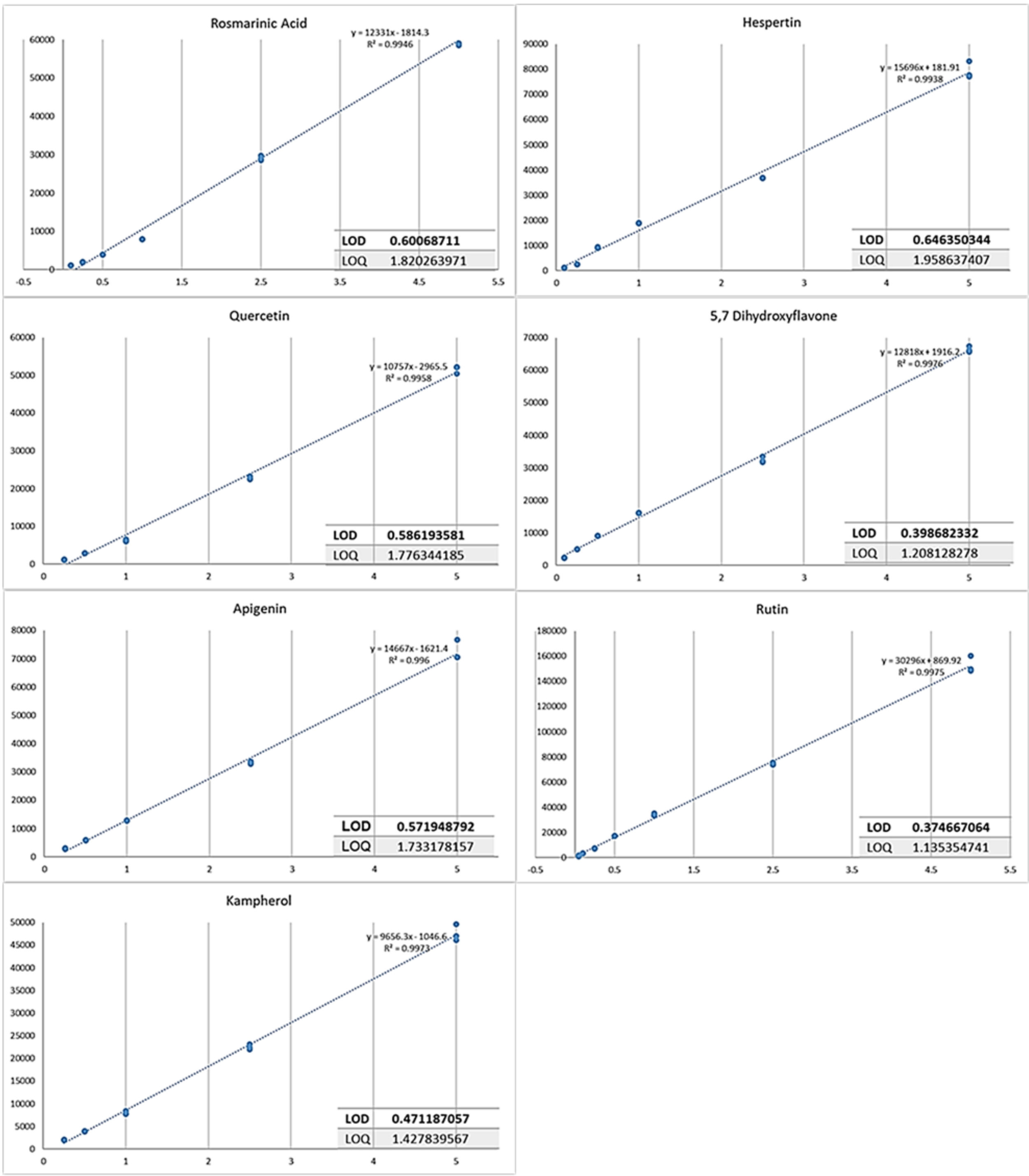

Figure S4b. Calibration Curves, LOD and LOQ for the selected Standards ((9) Rosmarinic acid, (10) Hesperidin, (11) Quercetin, (12) Apigenin, (13) Kaempferol, (14) Hesperetin, (15) Chrysin, (16) Rutin) with various ethanol concentrations for HPLC-UV analysis of the phenolic compounds in Ud extracts.

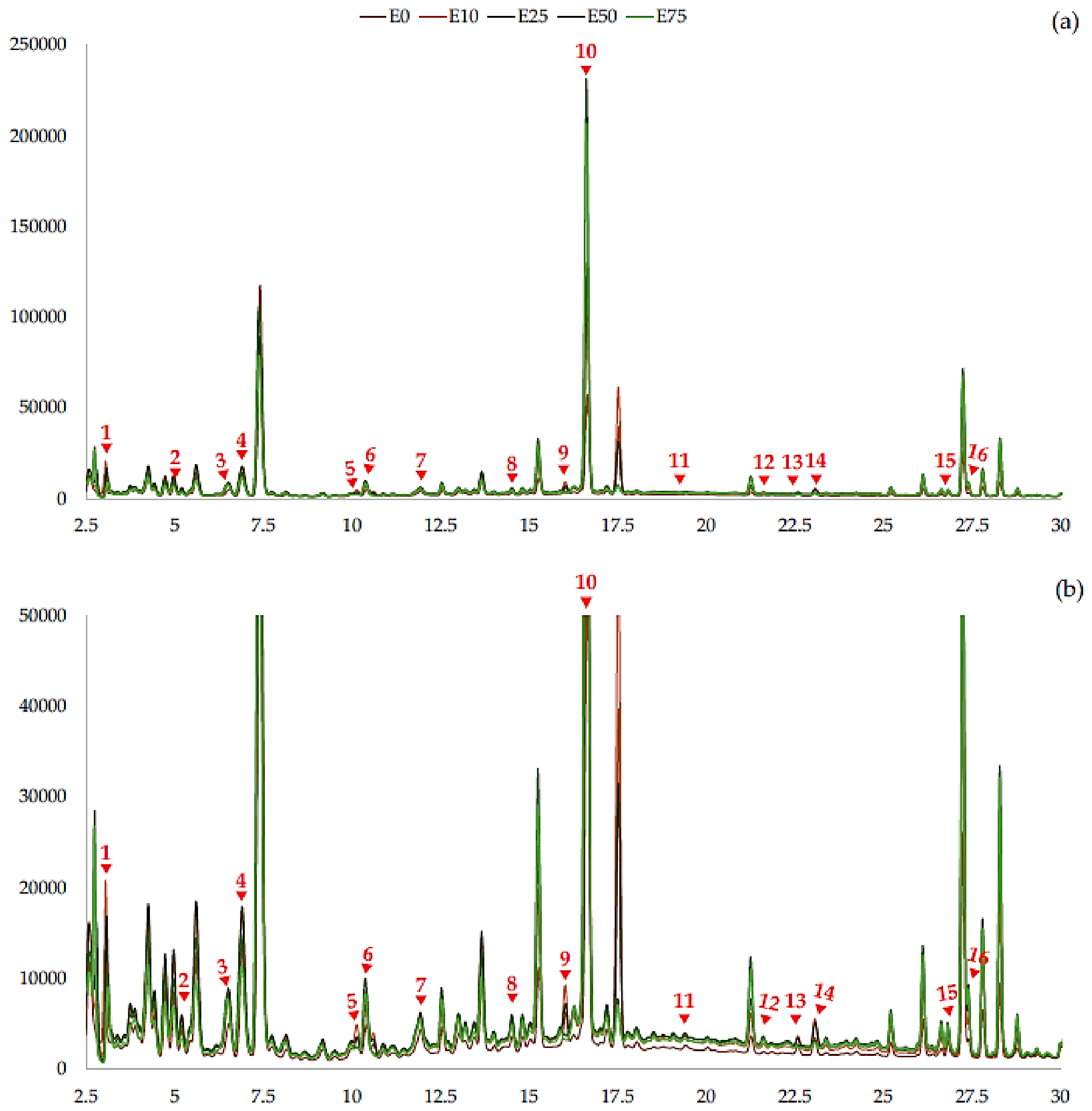

Figure S5. Example of chromatograms with variation in Ethanol concentration: (a) full-scale and (b) zoomed-in HPLC-UV chromatograms of the phenolic compounds in UACPE extracts with various ethanol concentrations. Annotations: (1) Gallic acid, (2) Chlorogenic acid, (3) Caffeic acid (4) Vanillic acid, (5) p-Coumaric acid, (6) Vanillin, (7) Ferulic acid, (8) 2-hydroxyccynnamic acid, (9) Rosmarinic acid, (10) Hesperidin, (11) Quercetin, (12) Apigenin, (13) Kaempferol, (14) Hesperetin, (15) Chrysin, (16) Rutin.

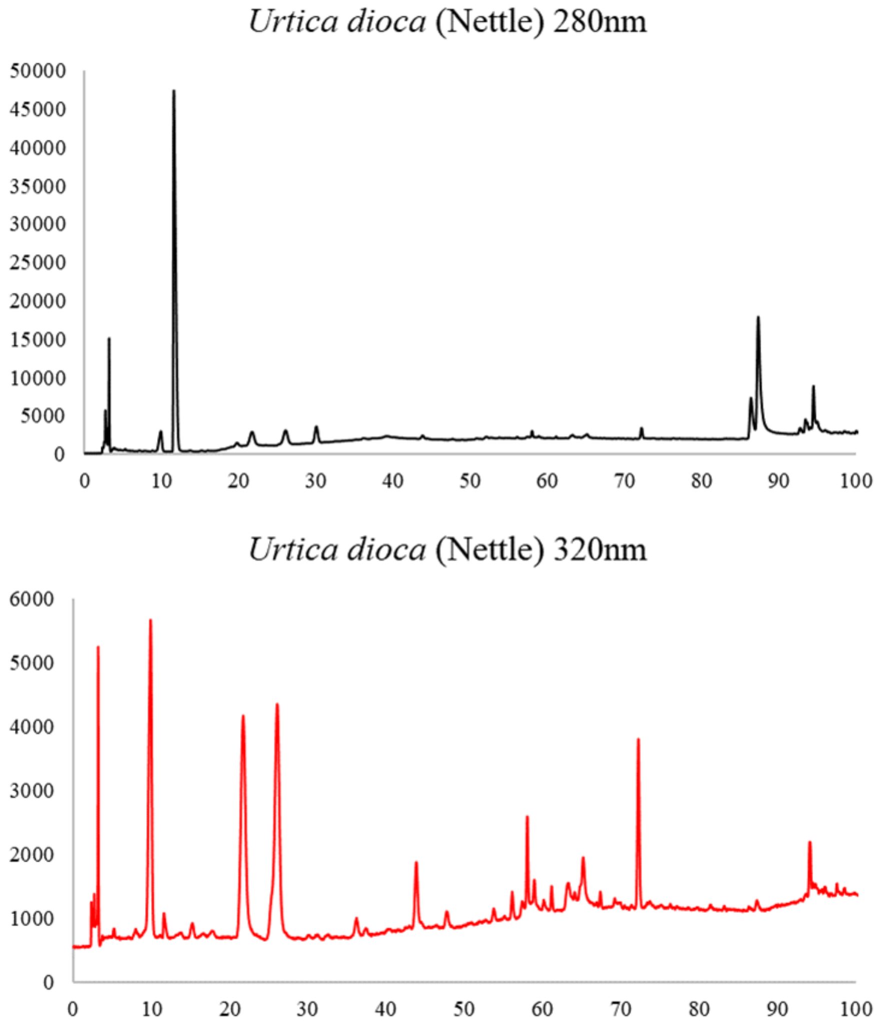

Figure S6. HPLC chromatographic profiles of UdE at 280 nm (A) and 320 nm (B). Peaks corresponding to key phenolic constituents are annotated: gallic acid (GA; tR = 3 min; LOD = 0.266 µg/mL; LOQ = 0.80 µg/mL), chlorogenic acid (CGA; tR = 12 min; LOD = 0.505 µg/mL; LOQ = 1.53 µg/mL), quercetin (Que; tR = 97 min; LOD = 0.586 µg/mL; LOQ = 1.77 µg/mL) and caffeic acid (CA; tR = 88 min; LOD = 0.600 µg/mL; LOQ = 1.82 µg/mL). Retention times (tR) are reported in minutes; LOD and LOQ values are reported as calculated for the method.

In the present study, the HPLC-based profiling provided a comprehensive chemical fingerprint of the extract, confirming that UdE is predominantly composed of phenolic acids (e.g., chlorogenic, gallic, caffeic, and rosmarinic acids) and minor flavonoids. This information is critical for contextualizing the antioxidant, antiviral, and immunomodulatory findings, as these classes of compounds are well-documented to influence redox balance, viral entry mechanisms, and inflammatory signaling pathways. Therefore, although additional experiments were not performed using isolated compounds, the compositional analysis supports a mechanistic interpretation at the extract level, consistent with a multi-target, synergistic mode of action.

Furthermore, the inclusion of phytochemical analysis aligns with standard practice in natural product research, where linking chemical composition to biological activity is essential for transparency, comparability across studies, and future reproducibility. It also enables meaningful comparison with the literature and provides a basis for future fractionation or compound-specific investigations.

References

1. Otles, S.; Yalcin, B. Phenolic compounds analysis of root, stalk, and leaves of nettle. Scientific World Journal 2012, **2012**, 564367. https://doi.org/10.1100/2012/564367
2. Orčić, D.; Francišković, M.; Bekvalac, K.; Svirčev, E.; Beara, I.; Lesjak, M.; Mimica-Dukić, N. Quantitative determination of plant phenolics in Urtica dioica extracts by high-performance liquid chromatography coupled with tandem mass spectrometric detection. Food Chem. **2014**, 143, 48–53. https://doi.org/10.1016/j.foodchem.2013.07.097
3. Dakhli, N.; López-Jiménez, A.; Cárdenas, C.; Hraoui, M.; Dhaouafi, J.; Bernal, M.; Sebai, H.; Medina, M.Á. Urtica dioica aqueous leaf extract: Chemical composition and in vitro evaluation of biological activities. Int. J. Mol. Sci. **2025**, 26, 1220. https://doi.org/10.3390/ijms2603122
4. Tlemcani, S.; Lahkimi, A.; Khibech, O.; Elrherabi, A.; Bouhrim, M.; Nasr, F.A.; Al-Zharani, M.; Qurtam, A.A.; Doubi, M.; Bekkari, H. Urtica dioica from El Menzel (Morocco): Phytochemical analysis, in vivo and in silico evaluation of analgesic and anti-inflammatory effects, and toxicological study with ADME profiling. Food Sci. Nutr. **2025**, 13, e71253. https://doi.org/10.1002/fsn3.71253
5. Kregiel, D.; Pawlikowska, E.; Antolak, H. Urtica spp.: Ordinary plants with extraordinary properties. Molecules **2018**, 23, 1664. https://doi.org/10.3390/molecules23071664.
